# Photocatalytic and Antimicrobial Properties of Cobalt-Doped Ag_3_PO_4_ Synthesized by Hydrothermal Method

**DOI:** 10.1021/acsomega.6c06289

**Published:** 2026-09-30

**Authors:** Daniel Pais Ospina, Yurimiler Leyet Ruiz, David Comedi, Ronaldo Santos da Silva, Simone Ramos de Castro, Jéssica Araújo Marques, Rubens Lucas de Freitas Filho, Marcos A. Bolson, Ézio Sargentini-Júnior, Lázaro Miranda Carvalho, Oscar Marin-Ramirez, Francisco Xavier Nobre

**Affiliations:** † Nanoproject − Laboratorio de Física del Sólido y Laboratorio de Nanomateriales, Departamento de Física, Facultad de Ciencias Exactas y Tecnología, 207899 Universidad Nacional de Tucumán, Avenida Independencia 1800, Tucumán CP 4000, Argentina; ‡ Instituto de Física del Noroeste Argentino (INFINOA), Consejo Nacional de Investigaciones Científicas y Técnicas (CONICET) y Universidad Nacional de Tucumán, Avenida Independencia 1800, Tucumán CP 4000, Argentina; § Laboratório de Processamento de Materiais Tecnológicos (LPMAT), Departamento de Ciências e Engenharia de Materiais, 67892 Universidade Federal do Amazonas, Manaus, Amazonas 69067-005, Brazil; ∥ Department of Physics, Federal University of Sergipe, São Cristóvão, Sergipe 49100-000, Brazil; ⊥ Departamento de Química, Alimentos e Meio Ambiente (DQA), 119525 Instituto Federal de Educação, Ciência e Tecnologia do Amazonas, Campus Manaus Centro (CMC), Manaus, Amazonas 69020-120, Brazil; # Grupo de Recursos Energéticos e Nanomateriais (GREEN), Instituto Federal de Educação, Ciência e Tecnologia do Amazonas, Campus Manaus Centro (IFAM-CMC), Manaus, Amazonas 6975-351, Brazil; ∇ Centro Multiusuário Para Análise de Fenômenos Biomédicos da, Universidade do Estado do Amazonas − CMABio, Manaus, Amazonas 69065-001, Brazil; ○ Departamento de Química, ICEx, Universidade Federal de Minas Gerais, UFMG, Belo Horizonte, Minas Gerais 31270-901, Brazil; ◆ 191073 Instituto Nacional de Pesquisas da Amazônia, INPA, Manaus, Amazonas 69084-000, Brazil; ¶ Instituto Federal de Educação, Ciência e Tecnologia do Piauí, IFPI, Campus José de Freitas, José de Freitas 64110-000, Brazil

## Abstract

Pristine and cobalt-doped silver phosphate (Ag_3_PO_4_) containing nominal Co concentrations of 0.5, 1, 3, and 5 mol % were synthesized by a hydrothermal method to investigate the influence of Co incorporation on structural, optical, photocatalytic, antimicrobial, and antiphotocorrosion properties. X-ray diffraction and Rietveld refinement confirmed preservation of the cubic Ag_3_PO_4_ structure, while variations in lattice parameters, Raman modes, particle morphology, and surface chemical states indicated Co-induced local distortion and defect formation. EDX and ICP-OES confirmed the presence of cobalt, whereas XPS revealed modifications in the Ag and O surface environments. The fundamental optical bandgaps ranged from 2.37 to 2.46 eV, with additional sub-bandgap absorptions at 1.68 and 1.65 eV for AgP:3Co and AgP:5Co, respectively. Progressive photoluminescence quenching demonstrated that Co strongly modifies charge-relaxation pathways. Despite their low BET surface areas (0.953–5.608 m^2^ g^–1^), the doped materials displayed enhanced photocatalytic activity, showing that performance was not controlled by surface area alone. AgP:3Co exhibited the highest methylene-blue removal under simulated solar irradiation, reaching 93.26% after 20 min, with 120.26 × 10^–3^ min^–1^ and *t*_1/2_ = 5.76 min. Scavenger experiments identified photogenerated holes and hydroxyl radicals as the predominant oxidative species. After five reuse cycles, AgP:3Co retained approximately 87% activity and showed greater structural and morphological stability than pristine Ag_3_PO_4_. All samples also exhibited strain-dependent antimicrobial activity against reference strains and clinical isolates. Overall, 3 mol % Co provided the most favorable balance among defect chemistry, charge utilization, photocatalytic efficiency, antimicrobial activity, and resistance to photocorrosion.

## Graphical abstract

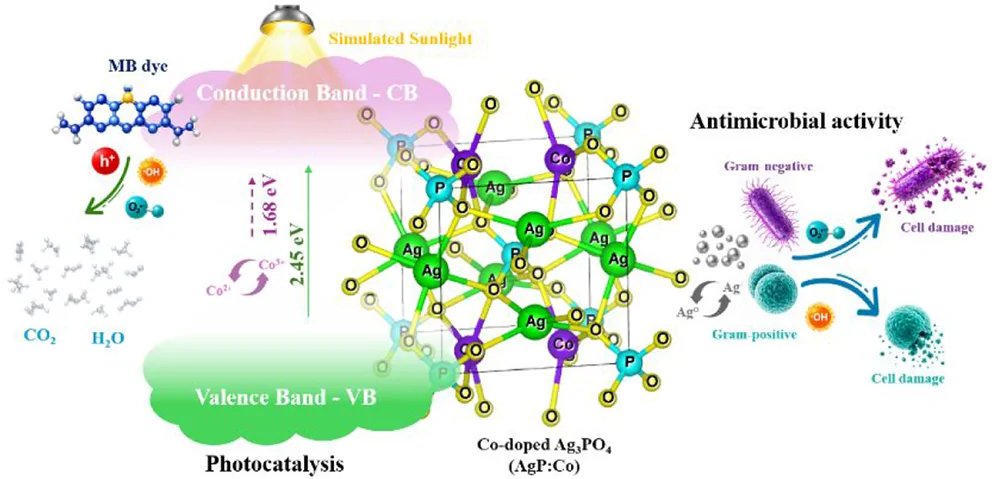

## 1 Introduction

Over the last few decades, materials science and solid-state physics have gained significant prominence due to their critical role in developing technological solutions for global challenges. The rapid industrialization and population growth have led to an alarming increase in the discharge of persistent organic pollutants and pathogenic microorganisms into water bodies. Consequently, the applications of semiconductors have expanded rapidly in fields such as chemical sensing, energy storage, hydrogen production, and environmental remediation.^1–3^ Regarding the latter, heterogeneous photocatalysis stands out as a sustainable and efficient advanced oxidation process (AOP) for the degradation of persistent organic pollutants and the inactivation of pathogenic microorganisms in aqueous media.^4^

To date, wide-bandgap semiconductors such as TiO_2_ and ZnO have been the most widely studied materials for environmental applications, owing to their high photoactivity, chemical stability, and low toxicity.^5–7^ However, these metal oxides are hindered by a fundamental limitation, their wide bandgap (>3.0 eV) is situated within the ultraviolet range. Given that UV light constitutes only approximately 4–6% of the solar spectrum, it is imperative to develop materials capable of harvesting the more abundant visible light region, which accounts for nearly 45% of solar energy.^8^ This constraint has driven the search for novel semiconductors with visible-light activation. While materials such as WO_3_,^9^ CuO,^10^ and CdS^11^ possess bandgaps in the visible range, they often exhibit lower photocatalytic performance compared to TiO_2_ or ZnO. In this context, silver phosphate (Ag_3_PO_4_) has emerged as a compelling semiconductor due to its narrow bandgap (∼2.4 eV), cost-effectiveness, and facile synthesis.^12–15^

Previous research has demonstrated that Ag_3_PO_4_ have outstanding photocatalytic activity, particularly in the degradation of organic dyes and water disinfection.^16,17^ Despite these advantages, the material faces a significant drawback: a high photocorrosion rate during the reaction which reduces the performance of this material when subjected to reuse cycles. Thus, under prolonged illumination, Ag^+^ ions tend to be reduced to metallic silver (Ag^0^), compromising the structural stability and recyclability of the material.^12^ To address this issue, various strategies have been explored, including the formation of heterostructures and transition metal doping.^18–20^

Several strategies have been proposed to improve the photocatalytic performance and mitigate the photocorrosion of Ag_3_PO_4_, including elemental doping,^21^ heterostructure formation,^4^ surface modification,^16^ and the deposition of cocatalysts.^12^ Doping with transition-metal or nonmetal ions can modify the electronic structure, introduce controlled defect states, and influence the separation and migration of photogenerated charge carriers.^15,22^ The construction of type-II, p–n, Z-scheme, or S-scheme heterostructures with other semiconductors can promote interfacial charge transfer, reduce electron–hole recombination, and limit the accumulation of electrons in the Ag_3_PO_4_ conduction band, thereby suppressing the reduction of Ag^+^ to Ag^0^.^23–25^ The effectiveness of these approaches depends on appropriate band alignment, interfacial contact, dopant concentration, and structural stability, since excessive defect formation or inadequate coupling may instead create recombination centers and impair photocatalytic activity.

Among these, the transition-metal doping has been extensively investigated as an effective strategy for tailoring the electronic structure and photocatalytic behavior of semiconductor materials. Previous studies involving Co-doped TiO_2_, ZnO, and other metal oxides have shown that the incorporation of Co^2+^ into the host lattice can introduce localized electronic states within the bandgap, extend light absorption toward the visible region, and modify the processes of photogenerated charge-carrier trapping and migration.^26,27^ In addition, Co-related defect states may influence the concentration of oxygen or cation vacancies required for charge compensation.^28^ At suitable dopant concentrations, these structural and electronic modifications can inhibit rapid electron–hole recombination and improve photocatalytic activity. Conversely, excessive Co incorporation may generate defect clusters or deep trapping states that act as recombination centers, resulting in a decline in photocatalytic performance.^29^ Therefore, the effect of Co doping strongly depends on its concentration, oxidation state, distribution, and interaction with the host lattice.

In Ag_3_PO_4_-based photocatalysts, the incorporation of transition-metal ions has been explored to control visible-light absorption, charge-carrier dynamics, and resistance to photocorrosion.^30–32^ For Co^2+^ doping, the substitution of monovalent Ag^+^ by divalent Co^2+^ requires a charge-compensation mechanism, potentially involving the formation of Ag vacancies and local distortions of the AgO_4_ and PO_4_ coordination environments. These changes may generate intermediate electronic states and alter the pathways of photoinduced electron transfer, thereby affecting both photocatalytic and antimicrobial performance. Thus, Co^2+^ ions may also contribute to the generation of reactive oxygen species through surface redox processes. Nevertheless, systematic investigations correlating Co content with the structural, surface-chemical, optical, photocatalytic, antimicrobial, and antiphotocorrosion properties of Ag_3_PO_4_ remain limited. This gap highlights the importance of evaluating a range of Co concentrations through complementary characterization and application assays.

In this study, pristine and Co-doped Ag_3_PO_4_ samples with nominal cobalt concentrations of 0.5, 1, 3, and 5 mol % were synthesized using a hydrothermal method. The effects of Co^2+^ incorporation on the physicochemical properties of Ag_3_PO_4_ were systematically investigated by X-ray diffraction (XRD) combined with Rietveld refinement, Raman spectroscopy, scanning and transmission electron microscopy (SEM and TEM), energy-dispersive X-ray spectroscopy (EDX), X-ray photoelectron spectroscopy (XPS), UV–Vis diffuse reflectance spectroscopy (DRS), photoluminescence (PL) spectroscopy, N_2_ adsorption–desorption analysis using the BET and BJH methods, and inductively coupled plasma optical emission spectrometry (ICP-OES). These complementary techniques enabled the evaluation of the structural, vibrational, morphological, textural, surface-chemical, compositional, and optical changes induced by cobalt doping. The photocatalytic activity and reusability of the materials were assessed through methylene blue degradation under simulated solar irradiation, while their antimicrobial activity was evaluated against different pathogenic bacterial strains using agar diffusion and serial microdilution assays.

## 2 Experimental Section

### 2.1 Materials

Silver nitrate – AgNO_3_ (Sigma-Aldrich, purity ≥ 99.98%), trisodium phosphate hydrated – Na_3_PO_4_•12H_2_O (Sigma-Aldrich, purity ≥ 97%), acetone—CH_3_COCH_3_ (Sigma-Aldrich, purity ≥ 99.5%), Methylene blue—C_16_H_18_ClN_3_S•xH_2_O (Sigma-Aldrich, purity ≥ 82%) and Cobalt­(II) nitrate hexahydrate—Co­(NO_3_)_2_•6H_2_O (Sigma-Aldrich, purity ≥ 99.9%), *p*-benzoquinone – C_6_H_4_(O = )_2_ (Sigma-Aldrich, purity ≥ 98.0%), tert-Butanol – (CH_3_)_3_-COH (Sigma-Aldrich, purity ≥ 99.5%), Lanthanum hexaboride – LaB_6_ (Sigma-Aldrich, 10 μm, purity ≥ 99.5%), and Ammonium oxalate monohydrate - (NH_4_)_2_C_2_O_4_•H_2_O (Sigma-Aldrich, purity ≥ 99.0%), were used without prior treatment. The double-distilled water used in the synthesis procedure had a pH of 5.2 and a conductivity close to 7.4 μS/cm.

### 2.2 Ag_3_PO_4_ (AgP) Synthesis

The synthesis of pure silver phosphate was carried out by modifying the experimental conditions described by Takeno et al.^16^ Initially, 2.4595 g of AgNO_3_ were added to a plastic Falcon tube (50 mL capacity) and dissolved in 10 mL of deionized water under vortex stirring, while 1.8533 g of Na_3_PO_4_•12H_2_O were dissolved in another plastic Falcon tube with 10 mL of deionized water under the same experimental conditions. Then, the sodium phosphate solution was slowly added to the silver nitrate solution in a PTFE reactor under constant stirring for 10 min. Subsequently, 1 mL of acetone was added to the mixture, which was then subjected to hydrothermal treatment at 120 °C for 2 h. The reported steps in the synthesis are schematically demonstrated in Figure 1 and eqs 1–3.

1 $${\mathrm{3AgNO}}_{3}\left(s\right)\overset{H_{2}O}{\;⃗}{\;\mathrm{3Ag}}^{+}\left(\mathrm{aq}\right){{\;+\;\mathrm{3NO}}_{3}}^{‐}\left(\mathrm{aq}\right)$$

2 $${\mathrm{Na}}_{3}{\mathrm{PO}}_{4}\left(s\right)\overset{H_{2}O}{\;⃗}{\;\mathrm{3Na}}^{+}\left(\mathrm{aq}\right){{\;+\;\mathrm{PO}}_{4}}^{\mathrm{3‐}}\left(\mathrm{aq}\right)$$

**Figure 1:**
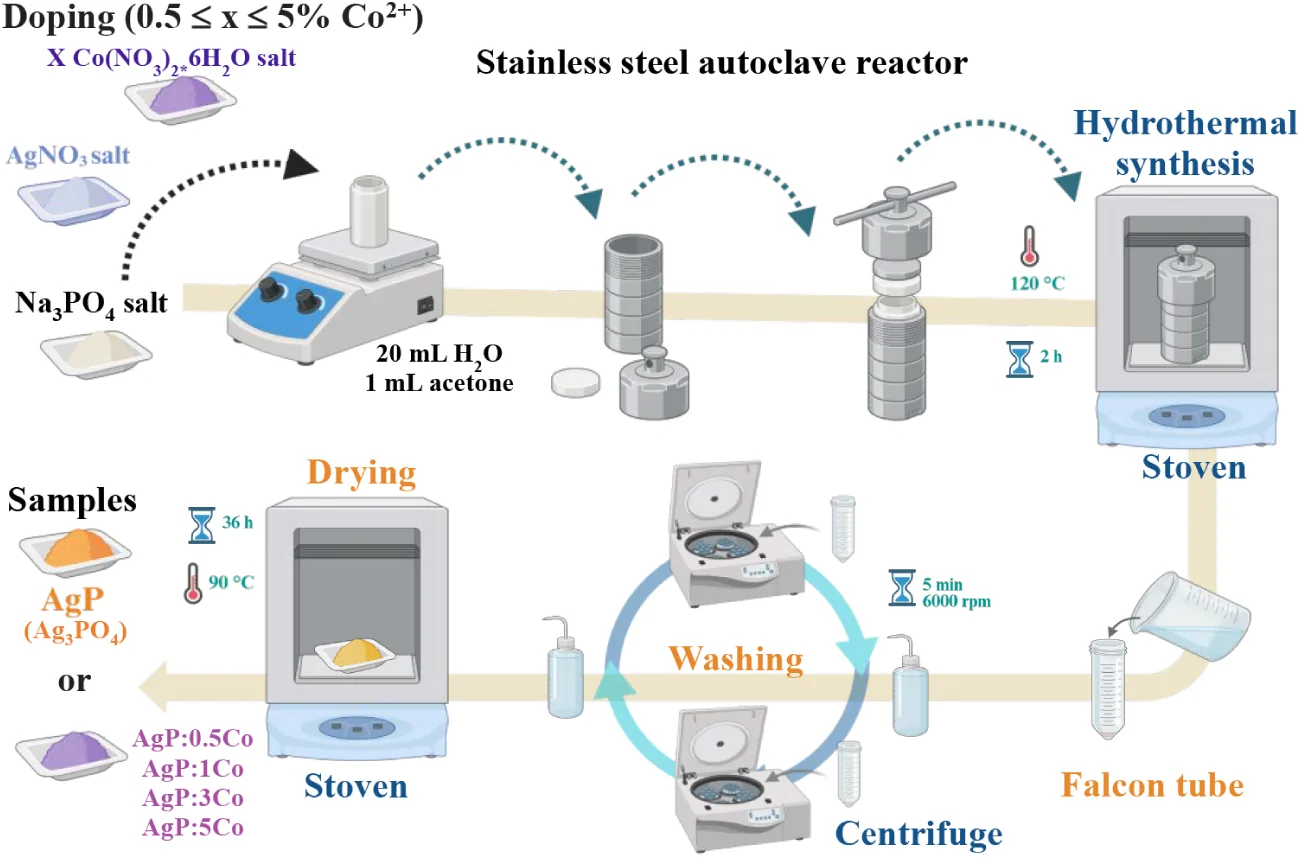
Synthesis steps in the hydrothermal synthesis of AgP and AgP:xCo samples.

Disregarding spectator ions, the reaction for obtaining silver phosphate becomes

3 $${\mathrm{3Ag}}^{+}\left(\mathrm{aq}\right){{\;+\;\mathrm{PO}}_{4}}^{\mathrm{3‐}}\left(\mathrm{aq}\right)\overset{HC/120^{◦}C}{\;⃗}{\;\mathrm{Ag}}_{3}{\mathrm{PO}}_{4}\left(s\right)$$

### 2.3 Co-Doped Ag_3_PO_4_ (AgP:xCo) Synthesis

Following the same procedure (Figure 1 and eqs 4–5), the cobalt-doped samples were synthesized by adding the appropriate amount of cobalt­(II) nitrate hexahydrate corresponding to the desired nominal doping percentages of *x* = 0.005 mol (AgP:0.5Co), 0.01 mol (AgP:1Co), 0.03 mol (AgP:3Co), and 0.05 mol (AgP:5Co). In this case, the silver ions were replaced by cobalt ions, with the original amount of silver ions that was used in the synthesis of pure silver phosphate, described in the previous paragraph. The proposed substitution is achieved through charge and mass balance, as described by eq 5

4 $$\mathrm{Co}{\left({\mathrm{NO}}_{3}\right)}_{2}•{6H}_{2}O\overset{H_{2}O}{\;⃗}{\;\mathrm{Co}}^{2+}\left(\mathrm{aq}\right){{\;+\;\mathrm{2NO}}_{3}}^{‐}\left(\mathrm{aq}\right){\;+\;6H}_{2}O\left(l\right)$$

Disregarding spectator ions, the reaction for obtaining cobalt doped silver phosphate becomes

5 $${\mathrm{xCo}}^{2+}\left(\mathrm{aq}\right)+\left(\mathrm{3‐2x}\right){\mathrm{Ag}}^{+}\left(\mathrm{aq}\right){{+\mathrm{PO}}_{4}}^{\mathrm{3‐}}\left(\mathrm{aq}\right)\overset{HC/120^{◦}C}{\;⃗}{\;\mathrm{Ag}}_{\mathrm{3‐2x}}{\mathrm{Co}}_{x}{\mathrm{PO}}_{4}\left(s\right)$$

### 2.4 Characterization

All detailed characterization procedures, as well as the experimental methodologies employed in the photocatalytic and antimicrobial assays, are provided in the Supporting Information. This supplementary document also contains additional experimental data, analytical parameters, and supporting results that complement the discussion presented in the main manuscript.

## 3 Results and Discussion

As presented in parts of Figure 2a–f, the characterization of pure and cobalt-doped silver phosphate was structurally performed via XRD, while structural details were investigated through Rietveld refinement.

**Figure 2:**
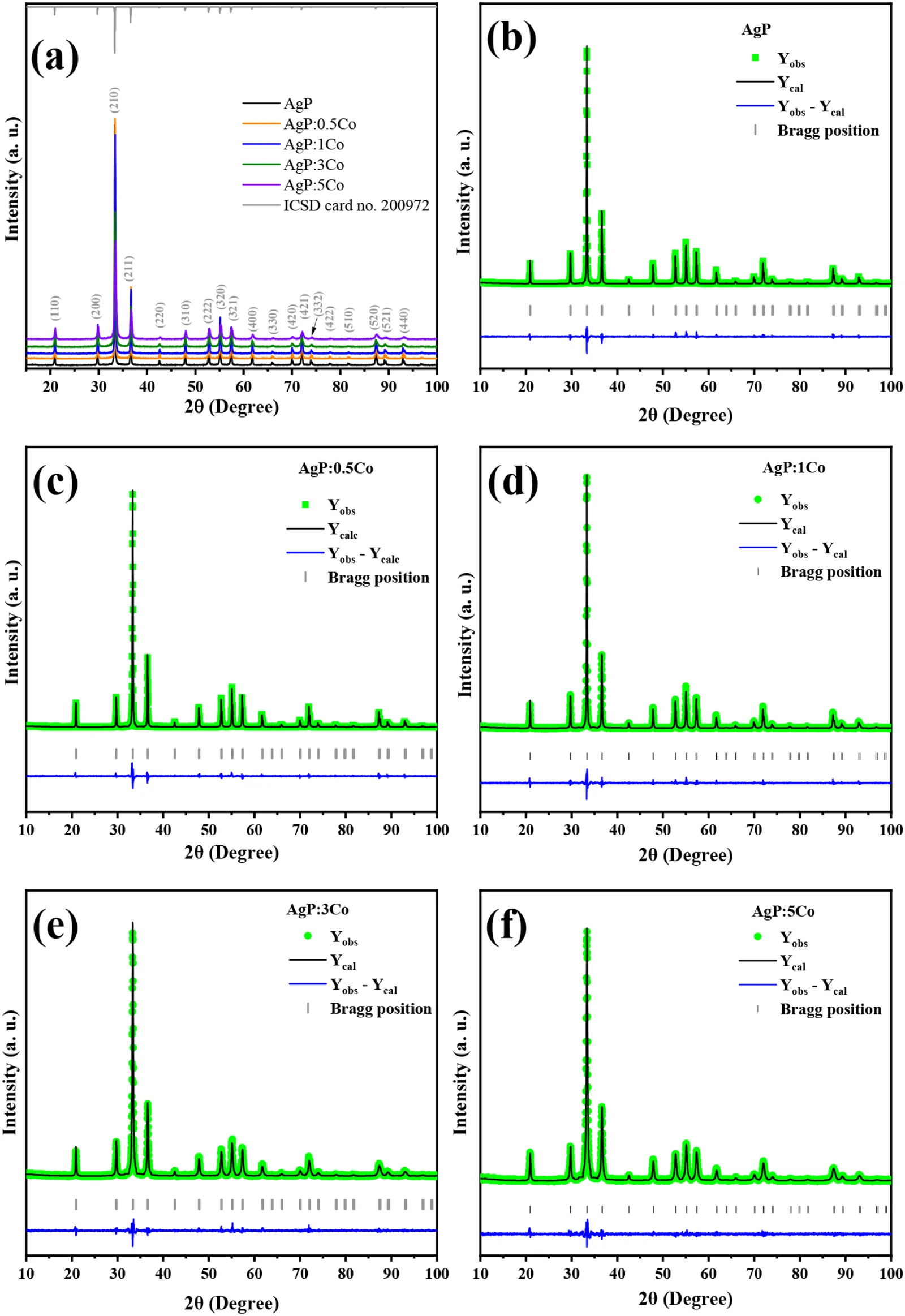
(a) Indexing for X-ray diffraction pattern of all samples, and Rietveld refinement plot of (b) AgP, (c) AgP:0.5Co, (d) AgP:1Co, (e) AgP:3Co, and (f) AgP:5Co samples.

As shown in Figure 2a, the diffraction peaks recorded over the range of 2θ = 15–100° were indexed to the cubic Ag_3_PO_4_ structure with the space group *P*4*®3n*. The main reflections were observed at approximately 2θ = 20.93° (110), 29.77° (200), 33.27° (210), 36.55° (211), 42.58° (220), 47.96° (310), 52.87° (222), 55.25° (320), 57.5° (321), 61.80° (400), 65.91° (330), 70.0° (420), 72.02° (421), 74.05° (332), 77.93° (422), 81.62° (510), 87.42° (520), 87.60° (520), 92.98° (440), and 98.33° (531). The positions and relative intensities of the experimental reflections are in good agreement with the crystallographic data reported for Ag_3_PO_4_ in the ICSD (entry No. 200972) and with previous literature reports.^12,33^

Furthermore, as seen in Figure 2a, no secondary phase diffraction planes or remaining reagents were identified in comparison to the standard diffraction pattern of Ag_3_PO_4_ from ICSD card No. 200972 (theoretical data), confirming that the proposed reaction for the formation of pure silver phosphate and the solid solutions was successfully achieved. Additionally, the graphical profile and intensity of the crystallographic planes observed for the synthesized samples are strong indicators of high crystallinity and short- and long-range organization throughout the crystal lattice,^24,34,35^

A detailed study of the structural parameters was conducted using the Rietveld method with the Fullprof Suite software (version Dec-2023), utilizing the individual experimental patterns and ICSD card 200972 as a reference parameters such as lattice constants (*a*, *b*, *c*, α, β and γ), atomic coordinates (*x*, *y* and *z*), unit cell volume (V_cell_) and crystallite size were analyzed. The spectra were refined using the Thompson-Cox-Hastings pseudo-Voigt* Axial divergence asymmetry function, with a sixth-degree polynomial for background adjustment.^16^ The quality of the fit was monitored via R-factors (R_p_, R_wp_, *R*_exp_) and the reduced chi-square (χ^2^), where values χ^2^ ≤ 2, indicate a good fit for the computed data.^4^

The structural refinement profiles shown in Figure 2b–f confirm the existence of crystallographic planes associated with a single crystalline phase (cubic of Ag_3_PO_4_), affirming the high purity of the prepared materials.^36^ The minimal residual observed in the Y_obs_ – Y_cal_ line implies that the experimental data (Y_obs_) are in excellent agreement with the calculated models (Y_cal_).

The lattice parameter results for each sample are summarized in Table 1. These data provide evidence of successful structure doping with cobalt ions, as a clear variation in the lattice parameters (*a* = *b* = *c*) is perceptible, ranging between 6.011(0)­Å and 6.006(5). A downward trend in these values is observed as a result of the insertion of, Co^2+^ (r^+^ = 65 – 75 pm) as substitutes for Ag^+^ ions (r^+^ = 126 – 129 pm). Consequently, the unit cell volume (V) for the silver phosphate decreased from 217.17(1) Å^3^ to 216.71(9) Å^3^, representing a reduction of approximately 0.21% due to the structural rearrangement of the solid solution.

**Table 1:** Structural Rietveld Refinement Results for the Lattice Parameters, Crystallite Size (D_hkl_), Unit Cell Volume (V) and Chi-Square for the Computed Data (χ^2^) for All Synthesized Samples and Standard Information from ICSD Card No. 200972^a^

	Samples					
Parameters	AgP	AgP:0.5Co	AgP:1Co	AgP:3Co	AgP:5Co	ICSD no. 200972
***a*** (Å)*	6.011(0)	6.007(0)	6.011(4)	6.008(0)	6.006(5)	6.009(6)
**α (°)** ^**#**^	90	90	90	90	90	90
*D_hkl_* **(nm)**	51(4)	51(3)	47(4)	33(2)	27(2)	-
**V(Å** ^**3**^)	217.17(1)	216.75(6)	217.23(5)	216.87(1)	216.71(9)	217.03
**χ** ^**2**^	1.65	1.72	1.74	1.49	1.36	-

a **Legend**: * Lattice parameters *a* = *b* = *c*; angle between lattices α = β = γ.

The contraction of the unit cell is consistent with the partial incorporation of the smaller Co^2+^ ions into Ag^+^ sites, as also supported by the compositional and surface-chemical results. Dopant-induced structural rearrangements have previously been reported for Ag_3_PO_4_ by Trench et al.,^37^ who observed changes in the P–O and Ag–O bond lengths and angles after the substitution of PO_4_^3 –^ groups by WO_4_^2 –^ ions. Although that study involved anionic-site substitution rather than the cationic substitution investigated here, it demonstrates that heteroionic incorporation into the Ag_3_PO_4_ lattice can induce local distortions and defect formation. Thus, the small and nonmonotonic variations in the lattice parameter and unit-cell volume observed in the present study is consistent with Co-induced structural modification of the Ag_3_PO_4_ host lattice.

Following structural refinement by the Rietveld method, the apparent crystallite sizes (D_hkl_) were estimated using the Scherrer equation.^38^ Instrumental broadening was evaluated from the diffraction pattern of a LaB_6_ standard reference material collected under the same instrumental conditions as the samples. The refined fwhm (Full width at half-maximum) values were corrected according to eq 6, where β_sample_ and β_LaB6_ correspond to the fwhm values of the sample and reference standard reflections from Lanthanum hexaboride (LaB_6_), respectively.

6 $$\beta_{corrected}=\sqrt[{2}]{\left({\beta_{sample}}^{2}-{\beta_{LaB_{6}}}^{2}\right)}$$

The corrected values were converted to radians and used in the Scherrer equation to calculate the apparent crystallite sizes by eq 7

7 $$D_{hkl}=\frac{k.\lambda }{\beta_{corrected}\cos\theta }$$

In this expression, *k* denotes the crystallite shape factor, for which a value of 0.91 was adopted based on the assumption of approximately spherical crystallites; λ is the wavelength of the Cu Kα radiation used for XRD data acquisition (λ_Cu_ = 0.15407 nm nm); β corresponds to the full width at half-maximum (fwhm) of the diffraction peak; and θ is the Bragg angle associated with the respective crystallographic plane. The calculation was performed using the average fwhm values obtained from the Rietveld-refined diffraction profiles corresponding to the (200), (210), and (211) planes, located at approximately 29.8°, 33.5°, and 36.8°, respectively.

According to Table 1, the *D_hkl_* values exhibit a decreasing trend as the structural doping with Co^2+^ions increases. The highest value recorded was *D_hkl_* = 51 ± 4 nm for the pristine silver phosphate, while the lowest value was observed for the AgP:5Co sample at *D_hkl_* = 27.4 ± 2.3 nm, representing a reduction of approximately 46.5%. This decrease is significant because crystallite size is a critical factor that directly impacts optical properties due to the quantum confinement effect. Furthermore, it influences morphological and textural properties, which serve as the foundation for applications in fields such as catalysis, antimicrobials, and energy storage or conversion,^4,13,34^

The vibrational and local structural properties of both pure silver phosphate (AgP) and cobalt-doped silver phosphate (AgP:0.5Co, AgP:1Co, AgP:3Co, and AgP:5Co) were investigated using Raman vibrational spectroscopy. This powerful analytical technique provides a deeper interpretation of the structural information obtained through X-ray diffraction.

Figure 3a–f show the Raman vibrational spectra for the samples. To provide detailed information regarding the position and intensity of the molecular vibration bands, a deconvolution of the Raman spectra was performed in the range between 85 cm^–1^ and 1100 cm^–1^.

**Figure 3:**
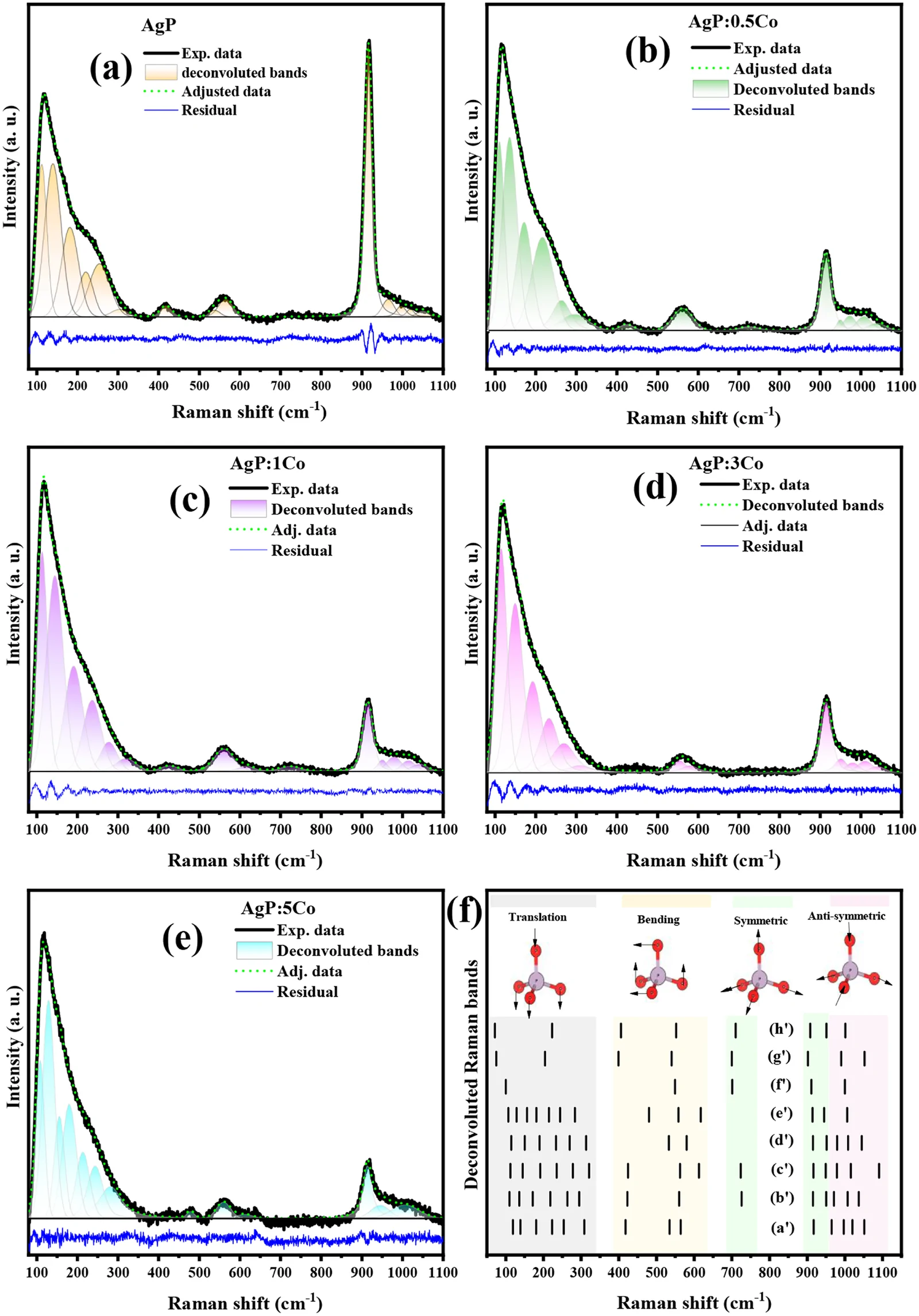
Deconvoluted Raman spectrum of (a) AgP, (b) AgP:0.5Co, (c) AgP:1Co, (d) AgP:3Co, (e) AgP:5Co, and (f) deconvoluted band positions versus Raman shift, where AgP (a’), AgP:0.5Co­(b’), AgP:1Co­(c’), AgP:3Co­(d’), AgP:5Co­(e’) and (f’), (g’) and (h’) are from Trench et al.,^36^ Almeida et al.^12^ and Santos et al.^24^.

According to Segundo Almeida et al.,^12^ cubic silver phosphate with the *P*4*3n* $\left(T_{d}^{4}\right)$ space group symmetry exhibits 18 active modes in Raman spectroscopy, which can be monitored in the range between 50 cm^–1^ and 1500 cm^–1^. In the present study, the reduced intensity or apparent absence of some Raman bands may result from a combination of spectral and material-related factors. Some vibrational modes of Ag_3_PO_4_ have intrinsically low Raman intensities and occur at closely spaced wavenumbers. Consequently, peak broadening and overlap may prevent these modes from being individually resolved within the spectral resolution and signal-to-noise ratio of the measurements. This effect is particularly relevant in the low-wavenumber region, where several translational and bending modes overlap. Spectral deconvolution was therefore employed to distinguish the underlying vibrational contributions.

In addition, the hydrothermal synthesis conditions and Co^2+^ incorporation can affect the short-range structural order of Ag_3_PO_4_. The substitution of Ag^+^ by Co^2+^, accompanied by Ag-site vacancies for charge compensation, may produce local lattice distortions, microstrain, and changes in the Ag–O coordination environment. These modifications can broaden the Raman bands, reduce their intensity, and cause small frequency shifts by altering local symmetry and phonon coherence. Variations in crystallite size, morphology, agglomeration, and dopant distribution may also influence Raman scattering. Therefore, the weakening or apparent disappearance of some components is interpreted as a consequence of band overlap and Co-induced local disorder rather than definitive evidence of the elimination of specific vibrational modes or the formation of secondary phases. Therefore, in agreement with the authors mentioned above, the irreducible formula for the active modes of silver phosphate is reported in eq 8

8 $${\Gamma =2A}_{1}{+4E+12T}_{2}$$

In this context, the A_1_ mode**s** are symmetric modes that do not undergo a change in symmetry due to molecular vibration. These are generally associated with the symmetric stretching of tetrahedral or octahedral clusters. In the specific case of silver phosphate, they are associated with the vibrations of the [PO_4_] clusters with tetrahedral symmetry.^35^ The E modes doubly degenerate, meaning there are two modes with the same vibration energy; therefore, they occur in two independent planes. Finally, the T_2_ modes are triply degenerate related to the spatial coordinates (x, y and z). In this case, they are associated with the stretching of the Ag–O or P–O bonds, as well as the oscillations of the angles within these bonds.^39^

As shown in Figure 3a, the Raman vibrational spectrum for the AgP sample bears a high resemblance to those reported in the cited literature,^12,24,36^ Consequently, a high-intensity band was identified at 919 cm^–1^ (A_1_), which is related to the symmetric stretching of the P–O bonds within the [PO_4_] units of tetrahedral symmetry. Lower intensity bands were also identified at 967 cm^–1^, 998 cm^–1^, 1022 cm^–1^ and 1053 cm^–1^, which may be related to T_2_ symmetry modes.^35^ The symmetry modes located at 565 cm^–1^ and 593 cm^–1^ are associated with the T_2_ symmetry mode, while the band at 418 cm^–1^ is related to E vibration mode.^36^ In the range between 80 and 368 cm^–1^, characteristic crystal lattice vibration modes are observed, as well as the free vibrations of the [PO_4_] units.^36,39^ In this study, active (deconvoluted) modes were identified at values of 112 cm^–1^, 140 cm^–1^, 123 cm^–1^, 222 cm^–1^, 255 cm^–1^ and 309 cm^–1^, all of which are associated with T_2_ vibration modes.

One of the primary modifications in the Raman vibrational spectrum of silver phosphate when doped with cobalt ions is a significant reduction in intensity of the symmetric vibration modes of the P–O bonds within the tetrahedral clusters. This behavior can be observed in the deconvoluted spectra for the samples AgP:0.5Co, AgP:1Co, AgP:3Co, and AgP:5Co, available in Figure 3b, c, d, and e, respectively. This trend may be related to structural disorder and the subsequent reduction in the degeneracy of the A_1_ modes due to the insertion of Co^2+^ ions into the crystal lattice as can be associated with the literature in Raman shift versus deconvoluted Raman band in Figure 3f. These ions possess a smaller ionic radius compared to Ag^+^ ions, leading to a reduction in both crystallite size and unit cell volume. These observations corroborate the results previously presented in the XRD analysis and Rietveld refinement.

An analogous behavior was observed by Trench et al.,^14^ who investigated the synthesis of silver phosphate doped with tungsten ions. Their study showed a gradual decrease in the intensity of the characteristic molecular vibration bands of silver phosphate, particularly of those located at 911 cm^–1^ (A_1_) and 100 cm^–1^ (T_2_), as a result of the substitution of [PO_4_] tetrahedral units by [WO_4_]. The authors noted that the doping process led to modifications in both optical and photocatalytic properties. These changes were attributed to the insertion of intermediate energy levels between the valence band (VB) and the conduction band (CB), modifications in surface energy (E_surf_) and an increased crystal lattice polarizability for the generation of oxidizing species, which resulted in a high oxidation rate of organic pollutant molecules (textile dyes) in an aqueous medium.

Figure 3f presents a schematic summary correlating the positions of the bands identified in the Raman spectra of samples AgP­(a’), AgP:0.5Co­(b’), AgP:1Co­(c’), AgP:3Co­(d’), and AgP:5Co­(e’). For comparison, the band positions identified as (f’), (g’), and (h’) were reported by Trench et al.,^36^ Almeida et al.^12^ and Santos et al.,^24^ all of which correspond to pure silver phosphate. Based on the graphical analysis, it can be inferred that doping with cobalt ions resulted in significant variations in the vibration modes associated with the symmetric and asymmetric stretching, as well as the distortions and translations of the [PO_4_] units. Therefore, these changes confirm the chemical doping of the silver phosphate structure.

Figure 4a–e presents the XPS survey spectra of pristine and Co-doped Ag_3_PO_4_ samples. All spectra contain the characteristic surface signals of Ag, P, and O, whereas Co-related signals are additionally detected in the doped materials.^19^ The main photoelectron signals are assigned to Ag 3d, P 2p, O 1s, and Co 2p, together with the corresponding Auger transitions. The C 1s signal observed at approximately 284.6 eV is attributed primarily to adventitious carbon adsorbed on the sample surfaces during handling and exposure to the atmosphere. No intense signals from unexpected foreign elements were observed, supporting the chemical purity of the surfaces within the detection limits of XPS.

**Figure 4:**
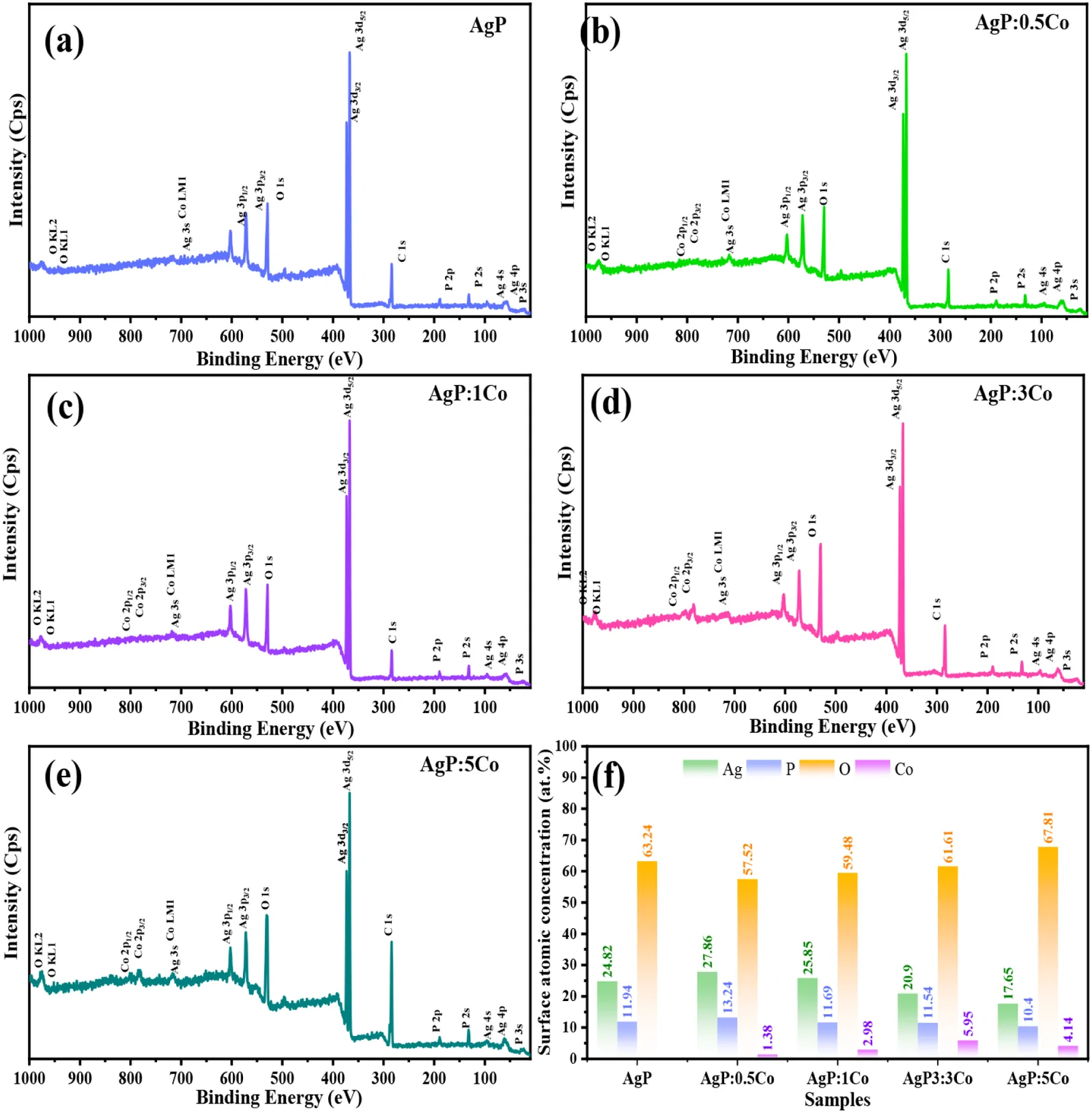
XPS survey spectra of (a) AgP, (b) AgP:1Co, (c) AgP:0.5Co, (d) AgP:3Co, and (e) AgP:5Co samples, showing the surface elemental signals associated with Ag, P, O, and Co. (f) Surface atomic concentrations (at.%) estimated from the XPS spectra using the corresponding relative sensitivity factors.

The surface atomic concentrations estimated from the survey spectra are summarized in Figure 4f. Pristine AgP exhibited surface concentrations of 24.82 at. % Ag, 11.94 at. % P, and 63.24 at. % O. After Co addition, cobalt was detected at the surfaces of all doped samples, confirming its presence within the XPS sampling depth. Nevertheless, the surface Co concentration did not increase monotonically with the nominal dopant content. In particular, AgP:3Co exhibited the highest surface Co concentration, whereas AgP:5Co showed a lower value. This behavior may be associated with a heterogeneous dopant distribution, differences in surface segregation, particle morphology, or the limited representativeness of a localized surface-sensitive measurement.^24^ The comparatively low Co contents also increase the uncertainty associated with their quantification from survey spectra.

At higher nominal Co concentrations, a relative reduction in the Ag surface concentration and an increase in the O contribution were observed. These changes suggest the development of an Ag-deficient and oxygen-rich surface, which may be associated with Ag-site vacancies required for charge compensation during the incorporation of divalent Co ions.^29^

Surface hydroxyl groups, adsorbed water, and other oxygen-containing species may also contribute to the O 1s intensity. Therefore, the total oxygen concentration determined by XPS cannot be attributed exclusively to lattice Oxygen.^40^

These surface-composition changes are consistent with Co-induced modification of the local chemical environment but do not, by themselves, demonstrate substitutional incorporation into the Ag_3_PO_4_ lattice. The high-resolution Ag 3d, P 2p, O 1s, and Co 2p spectra are discussed below to provide a more detailed interpretation of these surface chemical states.^19,36^

Figure 5 presents the high-resolution XPS spectra of Ag 3d, O 1s, P 2p, and Co 2p for pristine and Co-doped Ag_3_PO_4_.

**Figure 5:**
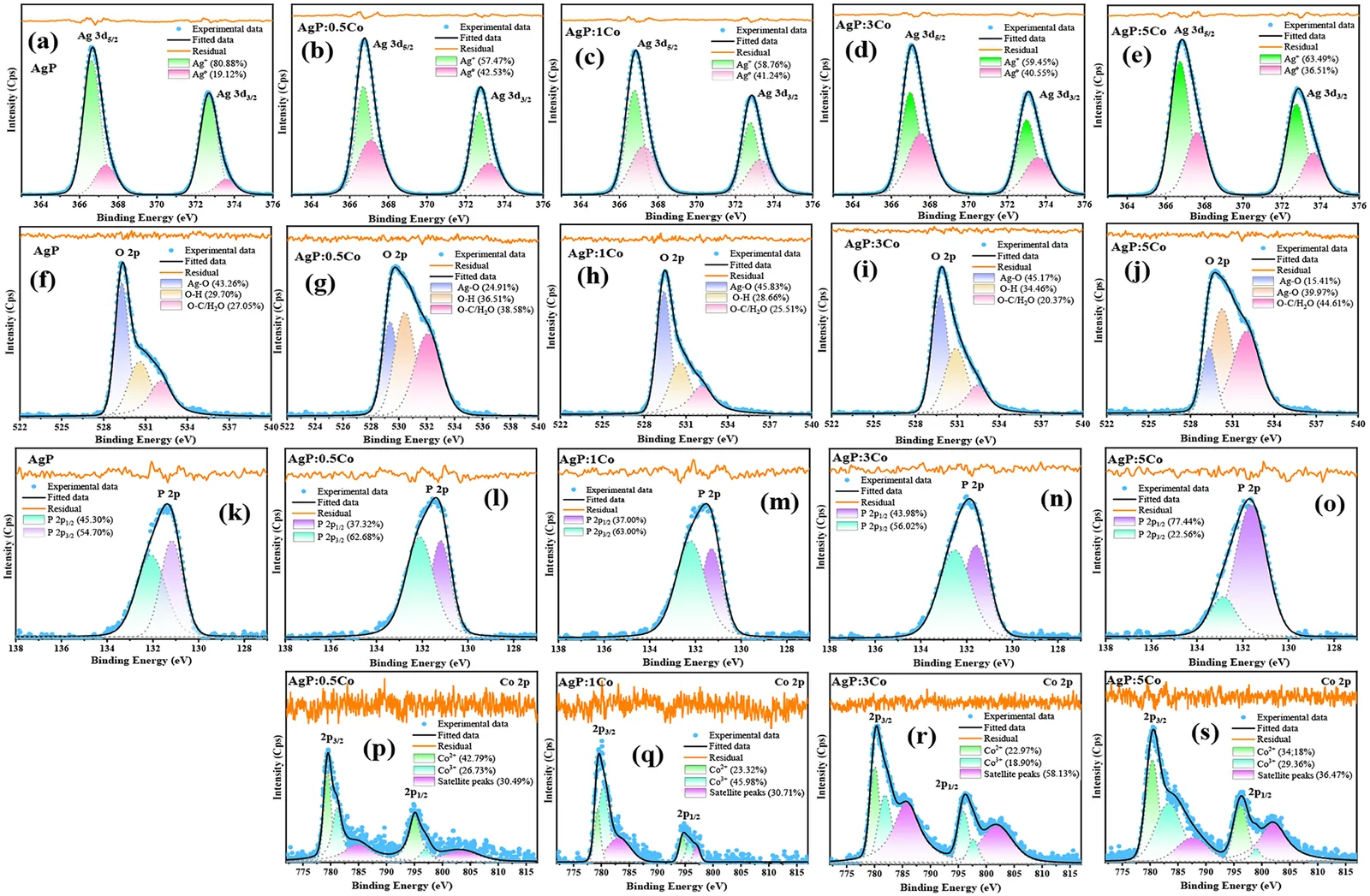
High-resolution XPS spectra and corresponding peak deconvolution of the synthesized samples: Ag 3d spectra of (a) AgP, (b) AgP:0.5Co, (c) AgP:1Co, (d) AgP:3Co, and (e) AgP:5Co; O 1s spectra of (f) AgP, (g) AgP:0.5Co, (h) AgP:1Co, (i) AgP:3Co, and (j) AgP:5Co; P 2p spectra of (k) AgP, (l) AgP:0.5Co, (m) AgP:1Co, (n) AgP:3Co, and (o) AgP:5Co; and Co 2p spectra of (p) AgP:0.5Co, (q) AgP:1Co, (r) AgP:3Co, and (s) AgP:5Co. Experimental data, fitted envelopes, residuals, individual chemical-state components, and shakeup satellite contributions are indicated in each panel.

The Ag 3d spectra in Figure 5a–e exhibit the characteristic spin–orbit doublet assigned to Ag 3d_5/2_ and Ag 3d_3/2_, with a separation of approximately 6.0 eV. Spectral deconvolution indicates the coexistence of Ag^+^ and Ag^0^ surface species in all samples, with Ag^+^ remaining the predominant contribution. The Ag^+^ component is associated with silver coordinated to oxygen in the Ag_3_PO_4_ lattice, whereas the lower-intensity Ag^0^ contribution indicates the presence of metallic silver at the particle surfaces.^41^ The latter may originate from partial reduction of Ag^+^ during synthesis, sample handling, exposure to light, or X-ray irradiation during XPS acquisition. Therefore, the Ag^0^ contribution should not be attributed exclusively to photocorrosion. The variation in the relative Ag^+^ and Ag^0^ contributions after Co addition demonstrates that cobalt modifies the surface chemical environment of silver.

The asymmetric O 1s spectra shown in Figure 5f–j were resolved into contributions associated with lattice oxygen in Ag–O/P–O environments and higher-binding-energy surface species.^42^ The lower-binding-energy component is attributed primarily to oxygen within the Ag_3_PO_4_ lattice, whereas the intermediate and higher-binding-energy components are associated with surface hydroxyl groups, adsorbed water, carbon–oxygen species, and other weakly bound oxygen-containing species.^43^ The relative increase in these surface-related components in some Co-doped samples indicates that Co incorporation modifies the surface coordination environment and favors the formation or adsorption of oxygenated species. This behavior is consistent with the oxygen-rich surface compositions identified from the survey spectra.^43^

The P 2p spectra in Figure 5k–o are characteristic of phosphorus in phosphate groups. The doublet is assigned to the P 2p_3/2_ and P 2p_1/2_ components of P^5+^ in $PO_{4}^{3-}$ . The absence of additional well-resolved P-related components indicates that phosphorus predominantly retains its phosphate chemical environment after Co incorporation,^24,32,44^ Small variations in the peak position or broadening may reflect changes in the local P–O coordination caused by lattice distortion, Ag-site vacancies, and modification of the neighboring Ag–O/Co–O environments.

These changes are consistent with the lattice-parameter variations observed by Rietveld refinement, although the magnitude of the shifts should be interpreted considering the experimental energy resolution and fitting uncertainty.

The Co 2p spectra of the doped samples are shown in Figure 5p–s. The profiles contain the Co 2p_3/2_ and Co 2p_1/2_ spin–orbit components together with pronounced shakeup satellite features.^45^ These satellites are characteristic of Co species with partially occupied 3d orbitals and provide strong evidence for the presence of Co^2+^.^46^

The spectral fitting also suggests a possible contribution from Co^3+^, indicating a mixed surface oxidation-state environment. However, because the Co concentration is relatively low and the Co 2p multiplet structure is complex, the calculated Co^2+^ /Co^3+^ proportions should be considered semiquantitative. The predominance of Co^2+^ supports the proposed substitution of Ag^+^ by Co^2+^, while the possible presence of Co^3+^ may be associated with surface oxidation and local charge-compensation processes.^47^

Overall, the high-resolution spectra demonstrate that Co addition modifies the chemical environments of Ag and O while preserving phosphorus predominantly as $PO_{4}^{3-}$ . When considered together with the XRD, Rietveld-refinement, ICP-OES, and EDX results, the XPS data support the presence of cobalt and Co-induced surface and structural modifications. However, because XPS probes only the near-surface region, these results should not be interpreted as direct quantitative evidence of the bulk stoichiometry.

The optical characterization was performed using spectroscopy in the ultraviolet and visible regions, adopting the diffuse reflectance method (UV–vis-DRS) in the range of 200 to 800 nm. Meanwhile, the optical bandgap (E_gap_) of the pure and the cobalt doped silver phosphate was obtained using the Kubelka–Munk method, as can be visualized in parts of Figure 6a–f.

**Figure 6:**
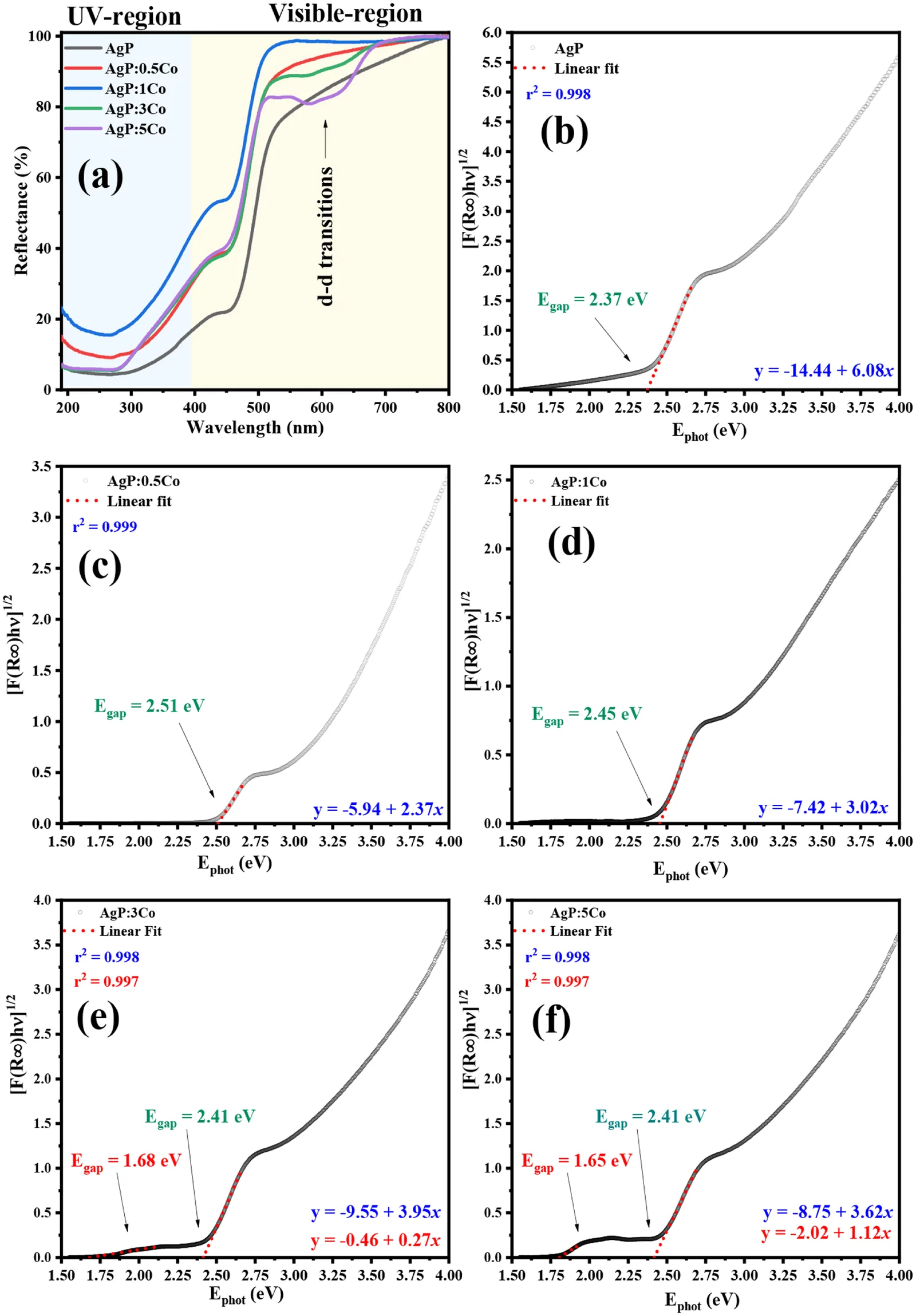
(a) Diffuse reflectance spectra of all samples and Tauc plots for (b) AgP, (c) AgP:0.5Co, (d) AgP:1Co, (e) AgP:3Co, (f) AgP:5Co samples.

As shown in Figure 6a, all samples exhibit pronounced light absorption in the visible spectral region, particularly within the wavelength range of 400–800 nm.^16^ According to the literature, this characteristics can be attributed to allowed indirect electronic transitions (*n* = 1/2),^4^ involving the O 2p and Ag 4d orbitals in the valence band (VB), while in the conduction band (CB), the Ag 5s and Ag 5p orbitals predominate. The reflectance spectra further reveals that the cobalt ion doping resulted in an increase of the reflectance compared to pure silver phosphate. This indicates that the density of states near the Fermi level predominantly involves the Co 3d (spin-up) states.^48^ Consequently, phosphorus and oxygen atoms also contribute to the valence band, while the top of the valence band and the bottom of the conduction band are governed by hybridized Co 3d and O 2p orbitals.^49^

The E_gap_ values of the samples were determined from the reflectance (R%) spectra as functions of the photon energy *E*_*phot*_, by plotting [*F*(*R*_∞_)*hν*]^n^ versus *E*_*phot*_ according to eq 9

9 $${[F\left(R_{\infty }\right)h\nu ]}^{n}=C_{1}\left(E_{phot}-E_{gap}\right)$$

where α corresponds to the Kubelka–Munk function,^48^ and C_1_ is a proportionality constant. Additionally, the value of *α* was obtained according to the mathematical formalism provided in eqs 10, 11 and 12,^50,51^

10 $$F\left(R_{\infty }\right)=\frac{{\left(1-R_{\infty }\right)}^{2}}{2R_{\infty }}=\frac{K}{S}$$

11 $$S=2R_{\infty }$$

12 $$K={\left(1-R_{\infty }\right)}^{2}$$

where R is the absolute reflectance, calculated by dividing the percentage reflectance values (%R) by 100, while K and S, are the molar absorptivity coefficient and the scattering coefficient, respectively.^18^ It is important to note that the exponent *n* in eq 9 depends on the type of electronic transition characteristic of the material. In this study, following the literature, a value of *n* = 1/2 was adopted, which corresponds to indirect allowed transitions^25^ consistent with reports from experimental studies correlated with Density Functional Theory (DFT) for the density of states (DOS).

Finally, the E_gap_ value for each sample was obtained by extrapolating the linear section of the curve from the plot of [*F*(*R*_∞_)*hν*]^1/2^ versus E_phot_ to α= 0. In this manner, the E_gap_ value for pure silver phosphate (AgP) was 2.37 eV, confirming the semiconductor properties of this material, whose main characteristic is the strong absorption of photons in the visible, in agreement with literature,^15,24,52^ The doping of AgP with cobalt ions at different percentages resulted in E_gap_ values of 2.51 eV (AgP:0.5Co), 2.45 eV (AgP:1Co), 2.41 eV (AgP:3Co), and 2.41 eV (AgP:5Co).

Despite the structural and morphological changes induced by Co incorporation, only modest variations were observed in the fundamental bandgap of Ag_3_PO_4_. The slightly higher values obtained for most Co-doped samples indicate that these modifications did not substantially alter the principal interband transition of the host semiconductor. However, AgP:3Co and AgP:5Co exhibited additional absorption features at lower energies, corresponding to approximately 1.68 and 1.65 eV, respectively (Figure 6e,f). These sub-bandgap features suggest the formation of Co-related localized electronic states within the Ag_3_PO_4_ band structure. Considering the electronic configuration of Co^2+^, these absorptions may be associated with transitions involving Co 3d states, although this assignment should be regarded as tentative. A similar behavior was reported by Hussien and Yahia^21^ for Ni-doped Ag_3_PO_4_ containing nominal Ni substitution levels from 1 to 30%. In that study, structural and morphological modifications were accompanied by two absorption regions: the fundamental absorption edge at approximately 2.43 eV and a secondary feature near 2.15 eV, which was attributed to electronic transitions involving the d orbitals of the transition-metal dopant. Thus, the present results indicate that Co incorporation has a limited influence on the fundamental bandgap of Ag_3_PO_4_ but introduces additional lower-energy optical transitions at higher dopant concentrations.

With the aim of investigating in detail the modifications to the optical and electronic properties of the samples, the valence (E_VB_) and conduction (E_CB_) band edge energies were calculated. For this purpose, the absolute Pearson electronegativity values (χ) for χAg, χO and χP of 4.44, 7.54, and 5.62 eV, respectively, were used. The electronegativity of silver phosphate $\left(\chi_{Ag_{3}PO_{4}}\right)$ was obtained from eq 13.^53^

13 $$\chi_{Ag_{3}PO_{4}}=\sqrt[{8}]{\chi Ag^{3}\times \chi O^{4}\times \chi P^{1}}$$

While the E_CB_ and E_VB_ were calculated from eqs 14–15.

14 $$E_{\mathrm{CB}}=\chi_{Ag_{3}PO_{4}}-E^{o}-0.5E_{\mathrm{gap}}$$

15 $$E_{\mathrm{VB}}{=E}_{\mathrm{CB}}{+E}_{\mathrm{gap}}$$

Where, *E* ^*o*^ corresponds to the free electron energy on the hydrogen scale (NHE), with an approximate value of 4.5 eV according to the literature.^54^ The results for all the samples studied are shown in Figure S1 (available in the Support Information). The samples with intermediate cobalt content have more negative E_CB_ values, as well as more positive E_VB_ values.

It should be noted that this model employs the absolute electronegativity of pristine Ag_3_PO_4_ and assumes an ideal semiconductor electronic structure. Therefore, the calculated values for the Co-doped samples should be regarded as approximate band-edge positions rather than direct experimental measurements. The incorporation of Co^2+^ may introduce localized defect states, alter the Fermi-level position, and modify the electronic environment near the band edges, effects that are not explicitly considered by this approach. Nevertheless, the relatively low Co concentrations and the preservation of the cubic Ag_3_PO_4_ crystalline structure, as indicated by XRD and Rietveld refinement, support the use of this method as a first-order approximation for a qualitative comparison among the samples.

The morphology and elemental composition of the synthesized samples were investigated by scanning electron microscopy (SEM) coupled with energy-dispersive X-ray spectroscopy (EDX), as shown in Figure 7a–j. Pristine Ag_3_PO_4_ (AgP) is composed predominantly of irregular and quasi-spherical particles with relatively smooth surfaces, together with some partially faceted particles in good agreement with the literature for Ag_3_PO_4_ synthesized by hydrothermal method,^36,53,55^ Considerable particle agglomeration and a broad size distribution are also observed. Therefore, the particles cannot be unequivocally described as well-defined cubes based on the available SEM images. Although XRD and Rietveld refinement confirm that Ag_3_PO_4_ crystallizes in the cubic structure, this crystallographic symmetry does not necessarily result in particles with an externally cubic morphology.^53,56^ Following Co^2+^ incorporation, changes in the apparent particle dimensions, degree of agglomeration, and morphological uniformity are observed, indicating that the dopant concentration influences the nucleation and crystal-growth processes. In general, the Co-doped samples exhibit smaller apparent particle sizes than pristine Ag_3_PO_4_, although the magnitude of this effect varies with the nominal Co concentration.

**Figure 7:**
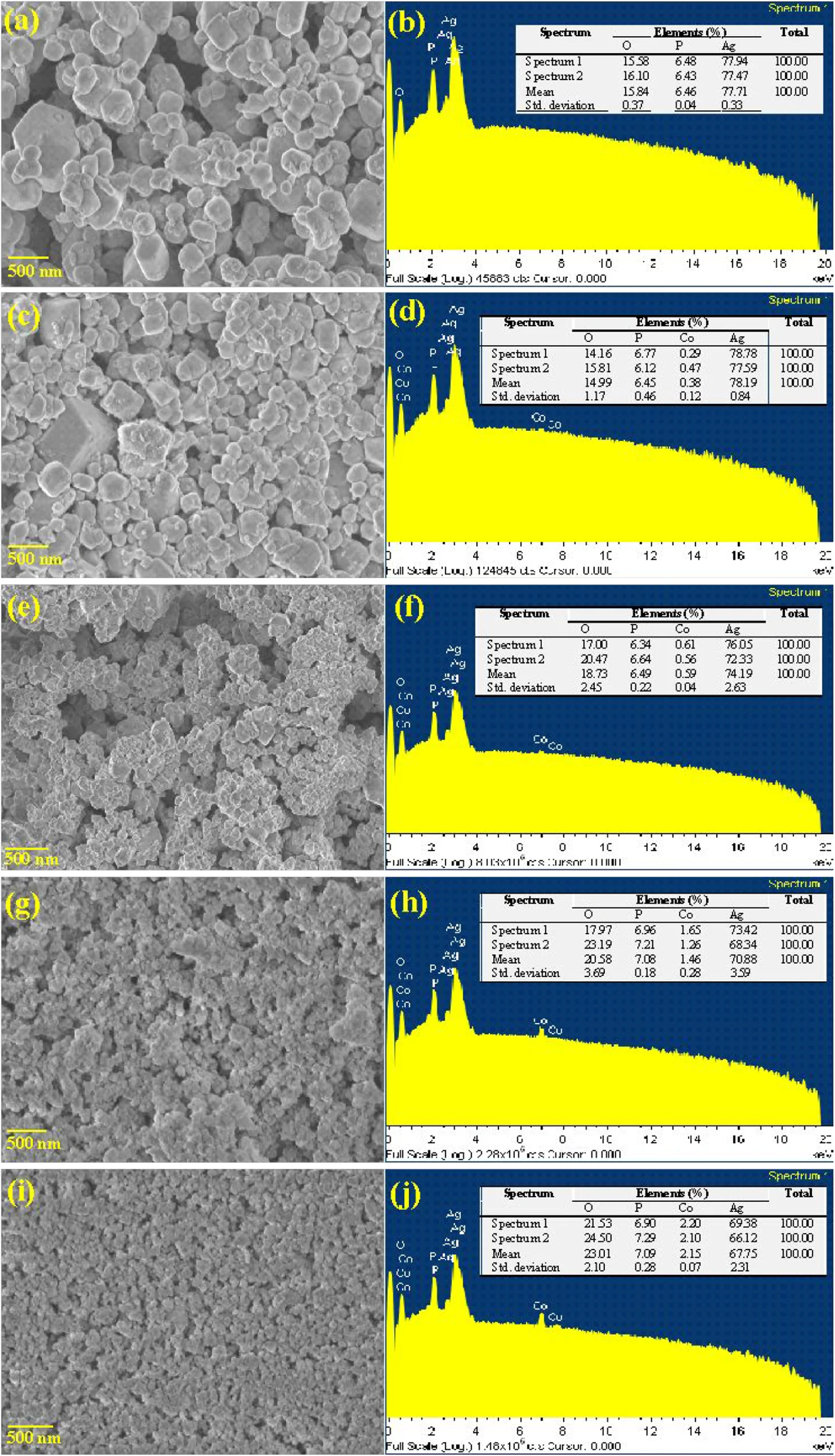
Scanning electron microscopy (SEM) and semiquantitative analysis by energy dispersive X-ray (EDX) for (a-b) AgP, (c-d) AgP:0.5Co, (e-f) AgP:1Co, (g-h) AgP:3Co and (i-j) AgP:5Co.

As the cobalt concentration increases from 0.5% to 5% mol, a clear transition from sub microsized cubic to quasi-spherical nanostructures is observed. The sample AgP:5Co exhibits the smallest average particle size (Figure S2), yet it shows the highest degree of agglomeration. The chemical composition and the successful integration of the dopant were verified through semiquantitative EDX analysis (Figure 7b-d-f-h-j). The spectra confirm the presence of silver, phosphorus, and oxygen in all samples, with the additional emergence of cobalt peaks in the doped series. For the AgP:5Co sample, the experimental atomic percentage of Co was found to be approximately 2.15 (wt %). This discrepancy between the nominal and experimental values suggests that a fraction of the cobalt ions might remain in the supernatant during the hydrothermal treatment.

Despite the varying cobalt content, the Ag:P atomic ratio remains close to the stoichiometric 3:1 ratio, indicating that the Ag_3_PO_4_ framework remains robust even under significant lattice distortion.^24^ These SEM observations are in excellent agreement with the Rietveld refinement data presented in Table 1. The reduction in the lattice parameter *a* from 6.011(0) Å to 6.006(5) Å and the decrease in crystallite size (D_hkl_) from 51.24 to 27.42 nm provide a solid crystallographic basis for the morphological changes seen in the SEM images. The contraction of the unit cell, caused by the smaller ionic radius of Co^2+^ (65–75 pm) compared to Ag^+^ (126–129 pm), acts as a driving force for the observed grain refinement.

The reduction in particle size, as confirmed by the distribution plots in Figure S2 (available in the Support Information), leads to a higher surface-to-volume ratio. To minimize the overall thermodynamic instability (surface Gibbs free energy), the smaller particles tend to coalesce, forming the dense clusters observed in the 3% and 5% Co^2+^ samples.

The elemental compositions determined by EDX and ICP-OES is presented in Table 2. Thus, based on these results, is confirmed the successful incorporation of cobalt into the Ag_3_PO_4_-based samples. As expected, Co was not detected in pristine AgP, whereas its concentration progressively increased with increasing nominal Co content. The Co concentrations obtained by EDX were 0.38, 0.59, 1.46, and 2.15 wt % for AgP:0.5Co, AgP:1Co, AgP:3Co, and AgP:5Co, respectively. A similar trend was observed by ICP-OES, which yielded corresponding values of 0.28, 0.59, 1.61, and 2.72 wt %. These results demonstrate that the amount of Co incorporated into the samples can be effectively controlled by adjusting its concentration during synthesis.

**Table 2:** Silver and Cobalt Contents in Pristine and Co-Doped Ag_3_PO_4_ Samples Determined by Energy-Dispersive X-Ray Spectroscopy (EDX) and Inductively Coupled Plasma Optical Emission Spectrometry (ICP-OES)^a^

	EDX		ICP-OES	
Sample ID	Ag (wt %)*	Co (wt %)*	Ag (wt %)*	Co (wt %)*
**AgP**	77.71 ± 0.33	ND	81.17 ± 0.08	ND
AgP:0.5Co	78.19 ± 0.84	0.38 ± 0.12	75.76 ± 0.02	0.28 ± 0.12
AgP:1Co	74.19 ± 2.63	0.59 ± 0.04	81.66 ± 0.03	0.59 ± 0.02
AgP:3Co	70.88 ± 3.59	1.46 ± 0.28	80.27 ± 0.05	1.61 ± 0.09
AgP:5Co	67.75 ± 2.31	2.15 ± 0.07	74.42 ± 0.08	2.72± 0.13

a The Results are Expressed as Weight Percentages (Wt%) and Presented as Mean ± Standard Deviation (SD). ND Indicates That the Element was Not Detected.

For pristine AgP, EDX indicated an Ag content of 77.71 ± 0.33 wt %, which is in excellent agreement with the theoretical Ag content of stoichiometric Ag_3_PO_4_ (approximately 77.31 wt %). ICP-OES yielded a slightly higher value of 81.17 ± 0.08 wt %, which may reflect differences in sample digestion, calibration, matrix effects, or the experimental uncertainties inherent to each analytical method. For the Co-containing samples, the Ag content determined by EDX generally decreased as the Co concentration increased, from 78.19 ± 0.84 wt % for AgP:0.5Co to 67.75 ± 2.31 wt % for AgP:5Co. This overall decrease is consistent with the progressive introduction of Co into the material and possible changes in the relative proportions of Ag-containing and Co-containing species.

The Ag concentrations obtained by ICP-OES ranged from 74.42 to 81.66 wt % and did not exhibit a strictly monotonic dependence on the nominal Co content. In particular, the relatively high Ag contents observed for AgP:1Co and AgP:3Co suggest that the incorporation of Co does not result in a simple proportional replacement of Ag throughout the entire compositional range. Instead, differences in incorporation efficiency, sample heterogeneity, defect formation, or the presence of minor Co-rich or Ag-rich regions may contribute to the observed variations. Therefore, these results alone cannot establish whether Co directly substitutes Ag^+^ in the Ag_3_PO_4_ lattice or is preferentially located at interstitial, defect-associated, or surface sites,^9,36,53^

The generally larger standard deviations observed by EDX, particularly for the AgP:1Co, AgP:3Co, and AgP:5Co samples, may indicate increasing local compositional heterogeneity at higher Co concentrations. This behavior is reasonable because EDX probes restricted regions of the particles and is considered a local and semiquantitative technique. In contrast, ICP-OES measures the average bulk composition after sample digestion and consequently provides more representative quantitative values for the overall elemental content. Despite the methodological differences, the comparable Co concentrations and their consistent increasing trend in both techniques provide strong evidence for the effective incorporation of Co into the synthesized materials.

Figure S3 (available in the Support Information) presents the N_2_ adsorption–desorption isotherms and the corresponding BJH pore-size distributions of pristine and Co-doped Ag_3_PO_4_. All samples exhibited low nitrogen uptake, consistent with their relatively low BET surface areas of 3.606, 1.511, 5.608, 4.681, and 0.953 m^2^ g^–1^ for AgP, AgP:0.5Co, AgP:1Co, AgP:3Co, and AgP:5Co, respectively. The surface areas followed the order AgP:1Co > AgP:3Co > AgP > AgP:0.5Co > AgP:5Co. This nonmonotonic behavior indicates that Co incorporation modifies the nucleation, crystal-growth, and aggregation processes but does not produce a proportional variation in accessible surface area.

The isotherm profiles are consistent with type IV behavior accompanied predominantly by H3-type hysteresis.^57^ The absence of a limiting adsorption plateau at high and the broad separation between the adsorption and desorption branches suggest capillary condensation within slit-shaped voids formed between irregular and aggregated particles.^58^ Therefore, the observed porosity is mainly attributed to interparticle spaces rather than to a uniform intrinsic pore network. This interpretation agrees with the SEM images, which revealed irregular morphologies, broad particle-size distributions, and substantial agglomeration.

The BJH pore-size distributions are concentrated predominantly in the small-mesopore region, particularly between approximately 2 and 5 nm, with lower-intensity contributions extending to approximately 10–15 nm. Isolated contributions at larger diameters, reaching approximately 35 nm, are likely associated with voids between particle aggregates rather than well-defined internal pores. AgP:3Co exhibits the strongest differential pore-volume contribution in the small-mesopore region, while AgP:1Co also shows a pronounced contribution, consistent with the higher N_2_ uptake and BET areas of these samples. In contrast, AgP:0.5Co and AgP:5Co display lower pore volumes and surface areas, indicating reduced accessibility of the interparticle spaces, possibly because of stronger aggregation.

Figure 8a–f presents the room-temperature photoluminescence spectra of pristine Ag_3_PO_4_ and the Co-doped samples. All materials exhibit a broad emission extending from approximately 420 to 750 nm, indicating the contribution of multiple and partially overlapping radiative transitions. Ag_3_PO_4_ has previously been reported to exhibit blue emission between 420 and 450 nm, commonly associated with electronic transitions involving clusters.^55^ Although this contribution is present in the current spectra, it is not the predominant emission. Instead, the spectra are dominated by broad green–yellow and red componentes.^53^

**Figure 8:**
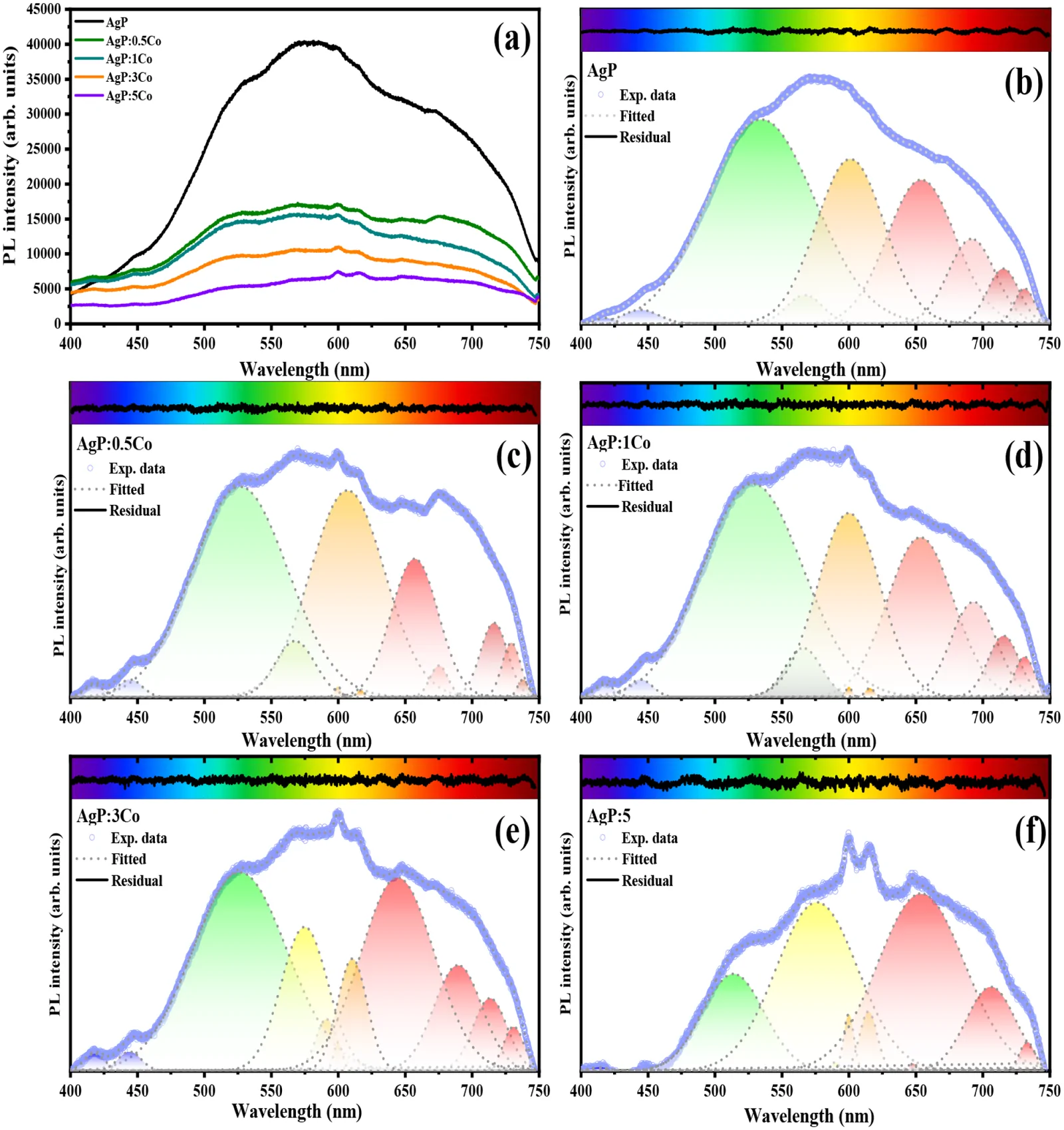
(a) PL spectra of AgP and Co-doped samples; deconvoluted emission bands of (b) AgP, (c) AgP:0.5Co, (d) AgP:1Co, (e) AgP:3Co, and (f) AgP:5Co.

The lower-energy emissions have frequently been associated with defect-mediated transitions involving local distortions of the coordination environment, Ag-site or oxygen-related defects, surface states, and partially reduced Ag^0^ species.^17^ Because these contributions overlap considerably, their assignments should be considered tentative rather than as unequivocal identification of individual defects.^36^

The deconvoluted spectra in Figure 8b–f reveal several components distributed across the blue, green, yellow, orange, and red regions. The higher-energy contributions may involve near-band-edge and shallow-defect transitions, whereas the yellow-to-red emissions are more consistent with recombination through localized states within the bandgap.^32^ These results agree with the XRD and Rietveld-refinement data, which demonstrate preservation of the cubic Ag_3_PO_4_ structure but reveal small variations in the lattice parameters and unit-cell volume after Co incorporation. Thus, although no secondary Co-containing crystalline phase was detected, the substitution of Ag^+^ by Co^2+^ and the associated formation of Ag-site vacancies may produce local lattice distortions that are not readily detected by conventional XRD but strongly influence the PL response.^59^ This interpretation is also consistent with the shifts, broadening, and intensity changes observed in the Raman bands associated with the Ag–O and P–O vibrational environments.

Moreover, the Co incorporation produces a pronounced and progressive reduction in PL intensity, following the general order AgP > AgP:0.5Co ≈ AgP:1Co > AgP:3Co > AgP:5Co. Although the overall broad emission profile is retained, the relative contributions of the deconvoluted bands change with Co content, indicating modification of the defect-state distribution and excited-state relaxation pathways. The DRS results showed only modest changes in the fundamental bandgap but revealed additional lower-energy absorption features for AgP:3Co and AgP:5Co. These features, with apparent energies of approximately 1.68 and 1.65 eV, respectively, are consistent with the formation of localized Co-related electronic states and may contribute to the red-region emissions. The XPS results, which indicate changes in the chemical environments of Ag and O and the presence of Co^2+^ /Co^3+^ surface species, further support the formation of Co-induced defect and surface states.

The PL-intensity trend does not follow the variation in particle morphology or BET surface area. The specific surface areas varied in the order AgP:1Co > AgP:3Co > AgP > AgP:0.5Co > AgP:5Co, whereas the PL intensity generally decreased with increasing Co content. Consequently, the quenching cannot be explained solely by particle-size reduction or increased surface area. Instead, it is primarily associated with Co-induced changes in the local coordination and electronic structure. Nevertheless, morphology, aggregation, and surface accessibility may affect the density and distribution of surface states and should therefore be considered complementary factors.

The photocatalytic performance of pure and cobalt-doped silver phosphate at different dopant concentrations was investigated through the degradation of methylene blue (MB) dye molecules under simulated visible light irradiation for 10 min of exposure. Accordingly, the experimental C/C_0_ ratio as a function of irradiation time for MB degradation using pure and cobalt doped silver phosphate is graphically presented in Figure 9a–h. In this case, *C* denotes the dye concentration at a given irradiation time, whereas *C*_*0*_ corresponds to the initial concentration of the solution, which was fixed at 10 mg L^–1^ in the present study.^4,37^

**Figure 9:**
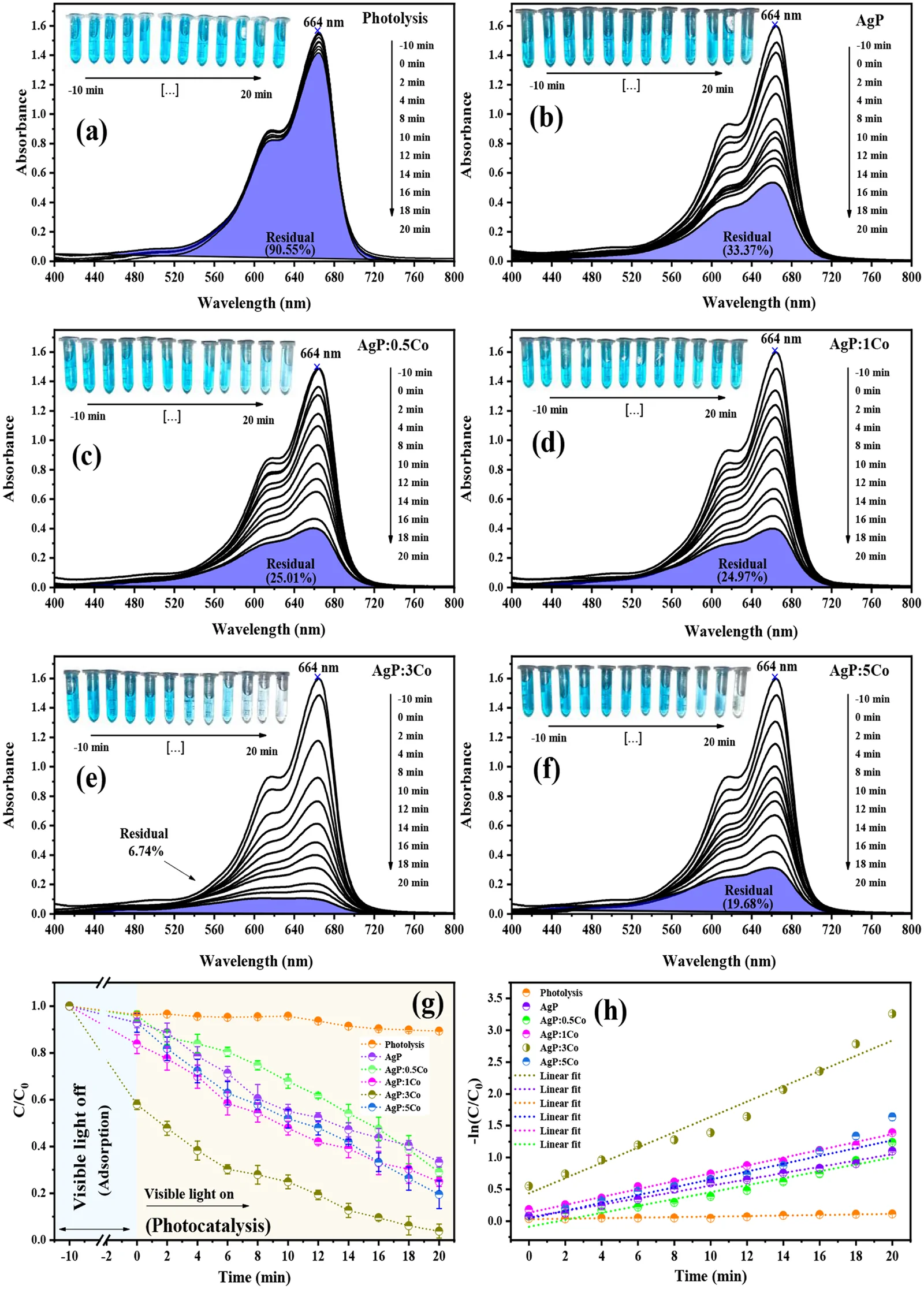
MB photodegradation under simulated solar irradiation: UV–Vis spectra for (a) photolysis, (b) AgP, (c) AgP:0.5Co, (d) AgP:1Co, (e) AgP:3Co, and (f) AgP:5Co; (g) profiles; and (h) pseudo-first-order kinetic fits. Insets show the color evolution during 10 min of dark adsorption and 20 min of irradiation.

The MB exhibits its characteristic maximum absorption band at approximately 664 nm, which is associated with the chromophoric structure of the dye.^7,22^ In the photolysis experimente (Figure 10a), only a slight decrease in this band was observed after 20 min, with 90.55% of the initial concentration remaining in solution. This result corresponds to only 9.45% removal and demonstrates that direct photolysis makes a minor contribution under the adopted irradiation conditions.

**Figure 10:**
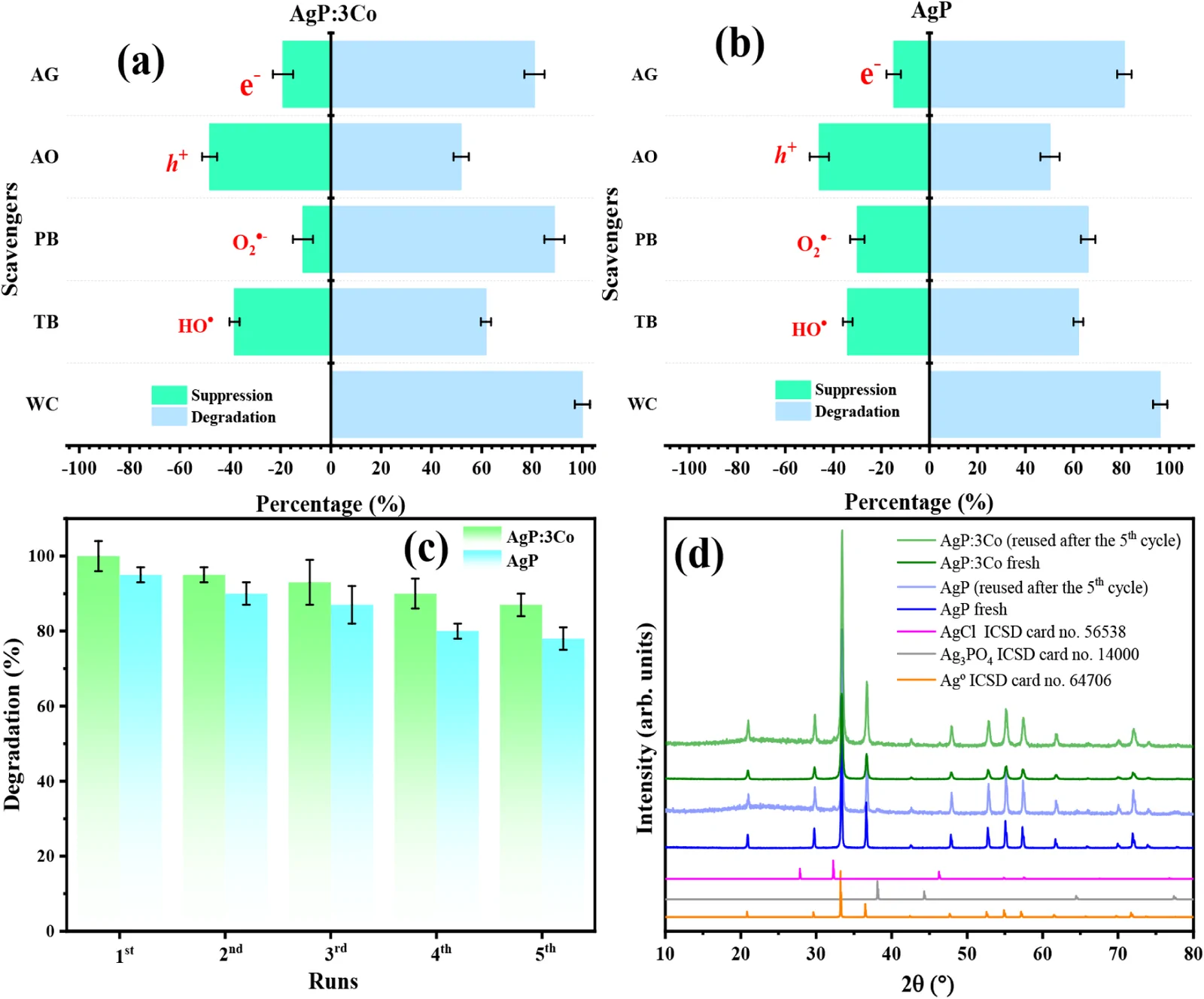
Reactive-species scavenging assays for (a) AgP:3Co and (b) AgP; (c) photocatalytic recyclability over five cycles; and (d) XRD patterns of fresh and reused catalysts after the fifth cycle, compared with reference patterns of Ag_3_PO_4_, AgCl, and Ag^0^.

In contrast, a continuous decrease in the absorption band at 664 nm was observed in the presence of all photocatalysts, confirming that Ag_3_PO_4_ promotes MB removal under simulated solar irradiation. After 20 min, the residual concentrations were 33.37%, 25.01%, 24.97%, 6.74%, and 19.68% for AgP, AgP:0.5Co, AgP:1Co, AgP:3Co, and AgP:5Co, respectively. These values correspond to overall removal efficiencies of 66.73%, 74.99%, 75.11%, 93.26%, and 80.55%. Therefore, all Co-doped samples exhibited greater overall MB removal than pristine Ag_3_PO_4_, with AgP:3Co displaying the highest performance.

The profiles in Figure 9g also reveal differences during the initial 10 min dark-equilibration period. AgP:3Co exhibited the most pronounced decrease in MB concentration before irradiation, indicating a greater contribution from adsorption. This behavior demonstrates that its overall removal efficiency results from both adsorption and photocatalytic degradation,^16,32,52^ Nevertheless, after the light source was turned on, AgP:3Co continued to exhibit the steepest decrease in C/C_0_ values, showing that its superior performance cannot be attributed exclusively to dark adsorption. The result likely reflects an optimum Co concentration that produces a favorable combination of surface adsorption, light absorption, defect-mediated charge transfer, and availability of reactive surface sites.

The improvement observed after Co incorporation may be associated with modifications in the local electronic and surface environments of Ag_3_PO_4_. At an appropriate concentration, Co-related states and Ag-site vacancies may participate in charge-carrier trapping and reduce the probability of immediate electron–hole recombination, increasing the availability of charge carriers for surface redox reactions,^14,16,18,21,41^

The XPS results also indicate changes in the Ag oxidation-state distribution and oxygen-containing surface species, which may affect the adsorption and activation of MB and dissolved oxygen. These interpretations are consistent with the structural, optical, and surface-chemical modifications identified by XRD, photoluminescence, diffuse reflectance, and XPS.^37,53^ However, the participation of specific reactive species should be established primarily from the radical-scavenger experiments rather than inferred from dye degradation alone.

Increasing the nominal Co content from 3 to 5 mol % resulted in a decrease in MB removal from 93.26% to 80.32%. This nonmonotonic behavior indicates the existence of an optimum dopant concentration. At higher Co contents, excessive defect formation, dopant aggregation, blockage of surface-active sites, or the generation of deep trapping states may favor nonradiative recombination rather than interfacial charge transfer. Consequently, although AgP:5Co remained more active than pristine AgP, its performance was inferior to that of AgP:3Co.

The plots of as a function of irradiation time are shown in Figure 9h. The approximately linear behavior indicates that MB removal can be described by an apparent pseudo-first-order kinetic model under the investigated conditions. The apparent rate constant (*k*_*app*_) associated with the oxidative processes involved in the photodegradation of the MB dye by pure and Co-doped silver phosphate microcrystals was determined using eq 16.^23,60^ In this equation, *t* represents the irradiation time, while *k*_*app*_ corresponds to the slope of the linear relationship obtained by plotting ln­(C/C_0_) as a function of the visible light exposure time.

16 $$\ln\left(\frac{C}{C_{o}}\right)=-k_{app}t$$

The slope of each linear fit corresponds to the apparent rate constant, and the kinetic order follows the general sequence AgP:3Co (120.26 × 10^–3^ min^–1^) > AgP:1Co (61.80 × 10^–3^ min^–1^) ≈ AgP:5Co (61.50 × 10^–3^ min^–1^) > AgP:0.5Co (54.47 × 10^–3^ min^–1^) > AgP (49.64 × 10^–3^ min^–1^) ≫ photolysis (4.17 × 10^–3^ min^–1^). This trend confirms that 3 mol % Co provided the most favorable balance of physicochemical properties for MB removal. Nevertheless, the reduction in the absorption band should be interpreted as dye decolorization or removal, since UV–Vis spectroscopy alone does not demonstrate complete degradation to CO_2_ and H_2_O.

In addition, as can be seen in the summarized results presented in Table 3, the half-life times calculated for the reactions considering the pseudo-first-order kinetic model were 5.76, 11.22, 11.27, 12.73, 13.96, and 166.22 min for the AgP:3Co, AgP:1Co, AgP:5Co, AgP:0.5Co, AgP, and Photolysis, respectively.

**Table 3:** The Percentage of Discoloration Rate (Dr), Apparent Rate Constants (*k_app_*), and Half Lifetime (*t*_1/2_), Final Time of Experiment (T), and Initial Concentration (C_0_) Obtained for Pure and Co-Doped Silver Phosphate Samples in the Present Study, Along with Representative Data Reported in the Previous Literature for Ag_3_PO_4_-based Photocatalysts Under Similar Conditions^a^

Sample	Dr (%)	*k*_app_ x 10^–3^ (min^–1^)	t (min)	C_0_ (mg L^–1^)	*t*_1/2_ (min)	ref.
**Photolysis**	10.68	4.17	20	10	166.22	TS
**AgP**	66.73	49.64	20	10	13.96	TS
AgP:0.5Co	74.99	54.47	20	10	12.73	TS
AgP:1Co	75.11	61.80	20	10	11.22	TS
AgP:3Co	93.26	120.26	20	10	5.76	TS
AgP:5Co	80.55	61.50	20	10	11.27	TS
**Ag**_**3**_ **PO**_**4**_	95	350.01	30	10	1.98	^31^
**Ag**_**3**_ **PO**_**4**_	56.51	4.74	120	50	146.20	^22^
**Ag**_**3**_ **PO**_**4**_	22	1.90	120	10	364.70	^17^
**Ag**_**3**_ **PO**_**4**_	46	9.20	60	10	75.34	^18^

a **Legend:** ref. = reference; TS = this study.

Considering the samples investigated in this study the AgP:3Co exhibited the highest photocatalytic efficiency, reaching 93.26% degradation of MB after only 20 min under simulated solar visible-light irradiation, with an apparent kinetic constant of 120.26 × 10^–3^ min^–1^ and a short half-life time of 5.76 min. Compared with previously reported Ag_3_PO_4_-based photocatalysts,^16–18,30^ AgP:3Co exhibited favorable photocatalytic performance, achieving 93.26% MB removal within 20 min, with an apparent rate constant of min^–1^ and a half-life of 5.76 min. Previous studies reported degradation efficiencies of 95% in 30 min (350.01 × 10^–3^ min^–1^), 56.51% in 120 min (4.74 × 10^–3^ min^–1^min^–1^), 22% in 120 min (1.90 × 10^–3^ min^–1^), and 46% in 60 min (9.20 × 10^–3^ min^–1^min^–1^). Although one reported system exhibited a higher apparent rate constant and a shorter half-life than AgP:3Co, the present catalyst outperformed the other referenced Ag_3_PO_4_ systems in terms of *k*_app_, half-life, and irradiation time required for substantial MB removal. Nevertheless, such comparisons should be interpreted cautiously because photocatalytic performance also depends on catalyst loading, dye concentration, irradiation spectrum and intensity, reactor geometry, and adsorption conditions.

Figure 10a,b presents the reactive-species trapping experiments for AgP:3Co and pristine AgP, respectively. The experiments were performed using silver nitrate (AG) as an electron scavenger, ammonium oxalate (AO) as a hole scavenger, *p*-benzoquinone (PB) as a superoxide radical scavenger, and *tert*-butyl alcohol (TB) as a hydroxyl radical scavenger. WC denotes the control experiment performed without any scavenger.

For AgP:3Co, the most pronounced suppression occurred after the addition of ammonium oxalate, followed by *tert*-butyl alcohol, indicating that photogenerated holes (*h* ^*+*^) and hydroxyl radicals (HO^•^) are the main oxidative species involved in MB degradation. The comparatively small inhibition produced by *p*-benzoquinone suggests a secondary contribution from superoxide radicals $\left(O_{2}^{•-}\right)$ , whereas the moderate effect of silver nitrate indicates that conduction-band electrons participate mainly through subsequent reduction reactions rather than through direct dye oxidation.

A similar trend was observed for pristine AgP, for which hole scavenging also produced the greatest reduction in photocatalytic activity, which is in well agreement with the reported by literature.^4,15,18,23,55,60^ However, *p*-benzoquinone caused substantially stronger suppression for AgP than for AgP:3Co, indicating a greater relative contribution of $O_{2}^{•-}$ radicals, in the pristine material. These differences suggest that Co incorporation modifies the charge-transfer and surface-reaction pathways. In AgP:3Co, Co-related states and Ag-site vacancies may favor hole-mediated surface oxidation and the formation of HO^•^ radicals, while decreasing the relative dependence on the electron-mediated reduction of dissolved oxygen. This interpretation is consistent with the PL quenching and XPS results, which indicate modification of the charge-relaxation pathways and surface oxygen environment after Co incorporation. Nevertheless, scavenger experiments provide indirect evidence, and possible effects of the scavengers on adsorption or reaction-medium chemistry should also be considered.

Figure 10c shows the recyclability of AgP and AgP:3Co over five consecutive photocatalytic cycles. AgP:3Co retained approximately 87% degradation efficiency after the fifth cycle, compared with approximately 78% for pristine AgP. The gradual decrease observed for both materials may result from catalyst loss during recovery, adsorption of reaction intermediates on active sites, particle agglomeration, and partial photocorrosion. However, the smaller activity loss and consistently higher performance of AgP:3Co demonstrate that the optimized Co content improves the operational stability of Ag_3_PO_4_. Co incorporation may reduce the accumulation of electrons at Ag^+^ sites and consequently limit, although not completely prevent, their photoreduction to Ag^0^.

The XRD patterns recorded before and after the fifth cycle are compared in Figure 10d. The principal reflections of the reused materials remain associated with the cubic Ag_3_PO_4_ phase, demonstrating that the host structure is largely preserved after repeated irradiation. Nevertheless, changes in peak intensity and the appearance or enhancement of minor reflections compatible with Ag^0^ and/or AgCl indicate partial surface transformation during photocatalysis. Metallic Ag may form through the photoreduction of lattice Ag^+^, whereas AgCl formation may occur through interaction between released Ag^+^ and chloride ions from the MB dye. These changes are more evident for pristine AgP, consistent with its greater loss of photocatalytic performance. AgP:3Co shows comparatively better preservation of activity and structural characteristics, supporting the conclusion that Co incorporation improves resistance to photocorrosion without completely suppressing surface-phase evolution.

The TEM images from Figure S4 (available in the Support Information) provide additional evidence of the morphological stability of AgP and AgP:3Co during photocatalytic reuse. The fresh samples exhibit irregular and strongly agglomerated particles, consistent with the SEM and BET results. After five cycles, both catalysts retain their aggregated morphology, although surface roughening, particle coalescence, and local restructuring are evident. These alterations appear more pronounced for pristine AgP, whereas AgP:3Co shows comparatively better preservation of its particle morphology. Together with the XRD and recyclability results, these observations suggest that Co incorporation improves the morphological stability and resistance of Ag_3_PO_4_ to photocorrosion.

Figure 11a–l presents representative agar-diffusion plates obtained for pristine and Co-doped Ag_3_PO_4_ against the reference strains and clinical isolate listed in Table 4. Figure 11a indicates the spatial distribution of the samples in each plate: positions 5, 6, 7, 8, and 9 correspond to AgP, AgP:0.5Co, AgP:1Co, AgP:3Co, and AgP:5Co, respectively. The formation of visible inhibition zones around the tested materials confirms their antimicrobial activity, while the differences in halo diameter demonstrate that the response depends on both Co concentration and bacterial strain.

**Figure 11:**
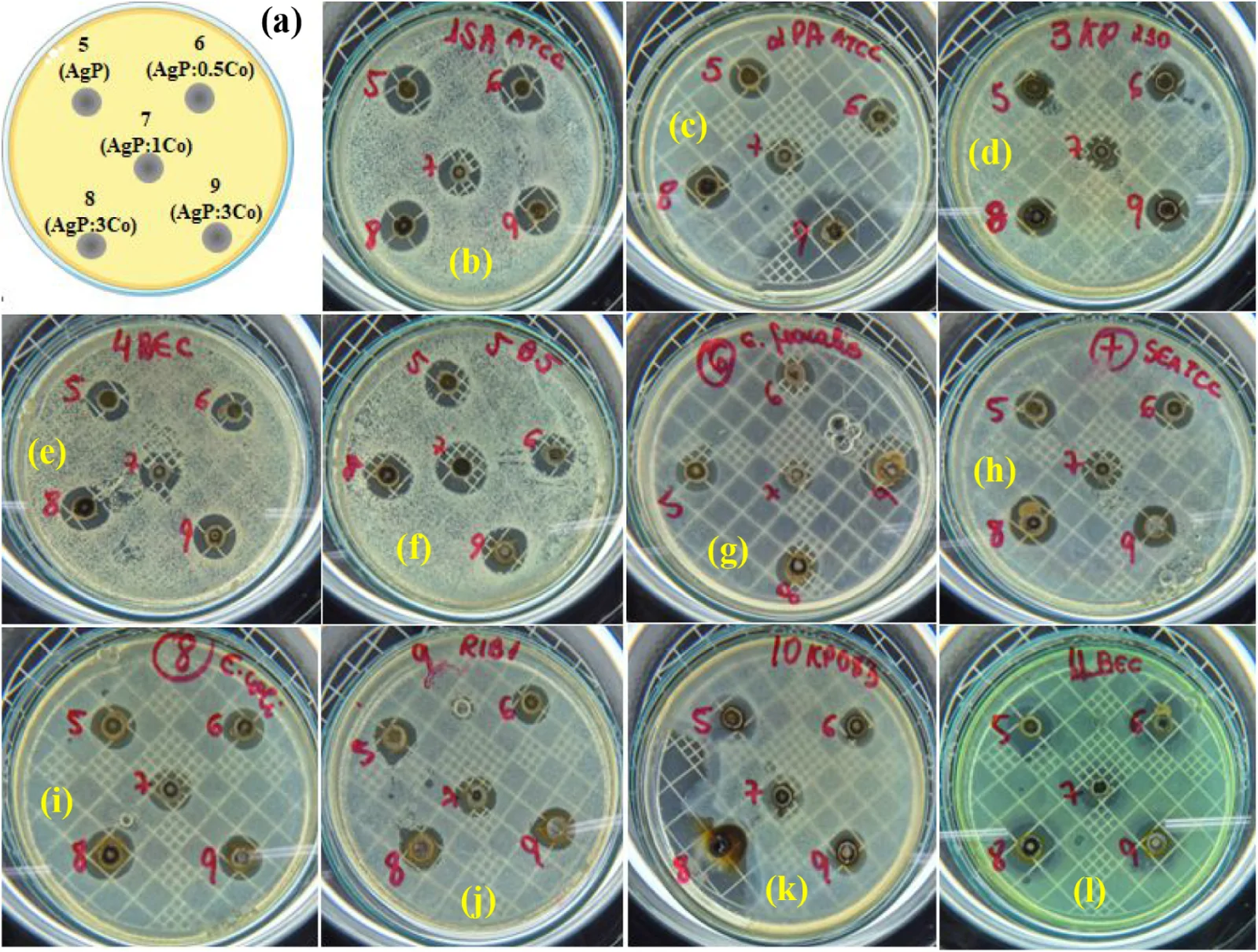
Agar-diffusion assays of pristine and Co-doped Ag_3_PO_4_: (a) sample-position guide (5: AgP; 6: AgP:0.5Co; 7: AgP:1Co; 8: AgP:3Co; and 9: AgP:5Co); (b) *S. aureus* ATCC 25922; (c) *S. aureus* CI 9393; (d) *E. faecalis* ATCC 19433; (e) *S. epidermidis* ATCC 12220; (f) *E. coli* ATCC 29214; (g) *K. pneumoniae* ATCC 4352; (h) *S. aureus* CI RIB1; (i) *P. aeruginosa* ATCC 27853; (j) *K. pneumoniae* CI 230; (k) *B. subtilis* ATCC 23857; and (l) *P. aeruginosa* CI 31 NM. CI: clinical isolate.

**Table 4:** Inhibition-Zone Diameters (mm) Obtained for Pristine and Co-Doped Ag_3_PO_4_ Samples Against Reference Bacterial Strains and Clinical Isolates Using the Agar Diffusion Method^a^ ^b^

	Tested Samples (mm)				
Bacteria	AgP	AgP:0.5Co	AgP:1Co	AgP:3Co	AgP:5Co
****Staphylococcus aureus*—ATCC 25922***	15 ± 0.01	15.5 ± 0.07	14.5 ± 0.21	15 ± 0.01	15.5 ± 0.07
****Staphylococcus aureus*—CI 9393***	15 ± 0.14	15 ± 0.14	13 ± 0.01	14.5 ± 0.07	13.5 ± 0.07
****Enterococcus faecalis* - ATCC 19433***	12.5 ± 0.07	12.5 ± 0.07	12 ± 0.01	13 ± 0.01	16.5 ± 0.49
****Staphylococcus epidermidis* - ATCC 12220***	14.5 ± 0.21	14.5 ± 0.21	14.5 ± 0.07	14 ± 0.01	14 ± 0.01
*Escherichia coli* ***ATCC – 29214***	13 ± 0.01	13.5 ± 0.07	13 ± 0.14	14 ± 0.14	14 ± 0.01
****Klebsiella pneumoniae* - ATCC 4352***	16 ± 0.01	13.5 ± 0.07	13.5 ± 0.20	13.5 ± 0.07	13.5 ± 0.07
****Staphylococcus aureus*—CI RIB1***	14.5 ± 0.21	14.5 ± 0.21	12.5 ± 0.07	13 ± 0.14	14 ± 0.01
****Pseudomonas aeruginosa* - ATCC 27853***	14 ± 0.14	14.5 ± 0.21	12.5 ± 0.07	13 ± 0.01	14.5 ± 0.21
****Klebsiella pneumoniae* - CI 230***	12 ± 0.14	13.5 ± 0.07	11.5 ± 0.07	12.5 ± 0.07	13.5 ± 0.07
****Bacillus subtilis* - ATCC 23857***	13 ± 0.28	16 ± 0.14	14 ± 0.42	14.5 ± 0.21	14 ± 0.28
****Pseudomonas aeruginosa* - CI 31 NM***	14.5 ± 0.07	16 ± 0.14	14.5 ± 0.07	16 ± 0.14	16.5 ± 0.21

a Values are Expressed as Mean ± Standard Deviation.

b **Legend:** CI = Clinical isolated; ATCC = American Type Culture Collection.

For *Staphylococcus aureus* ATCC 25922, all samples produced similar inhibition zones of 14.5–15.5 mm, indicating that Co incorporation caused only minor changes in activity. A comparable behavior was observed against *Staphylococcus epidermidis* ATCC 12220 and *Escherichia coli* ATCC 29214, for which the inhibition diameters remained within relatively narrow ranges. Conversely, a marked composition-dependent response was observed for *Enterococcus faecalis* ATCC 19433: AgP:5Co produced the largest inhibition zone (16.5 ± 0.49 mm), compared with 12.5 ± 0.07 mm for pristine AgP. AgP:0.5Co also showed enhanced activity against *Bacillus subtilis* ATCC 23857, increasing the inhibition diameter from 13.0 ± 0.28 to 16.0 ± 0.14 mm.

The clinical isolates also displayed distinct susceptibility patterns. For *Klebsiella pneumoniae* CI 230, AgP:0.5Co and AgP:5Co generated larger inhibition zones than pristine AgP, whereas AgP:3Co and AgP:5Co exhibited the strongest activity against *Pseudomonas aeruginosa* CI 31 NM, both reaching 16.5 mm. In contrast, pristine AgP was more effective against *K. pneumoniae* ATCC 4352 and *S. aureus* CI RIB1 than most Co-doped compositions. These results demonstrate that cobalt incorporation does not produce a monotonic or universal improvement in antimicrobial performance; instead, the optimal Co content depends on the tested microorganism.

The strain-dependent behavior may be related to differences in cell-wall organization, membrane composition, defense mechanisms against oxidative stress, and susceptibility to released metal ions. The antimicrobial action of these materials may involve contact-mediated membrane damage, release of Ag^+^ and Co species, and possible oxidative stress caused by reactive oxygen species. Co-induced changes in particle morphology, surface chemical states, Ag-site vacancies, and oxygen-containing surface species, as indicated by SEM, XPS, and BET analyses, may modify the interactions between the materials and bacterial cells.

The data presented in Table 5 and Figure S5 demonstrate the antimicrobial performance of pure silver phosphate (AgP) and cobalt-doped silver phosphate (AgP:xCo) against different Gram-positive and Gram-negative bacteria, enabling a comparative analysis both among formulations and among microorganisms. This pattern suggests an antimicrobial effect typical of silver-based materials, in which bactericidal activity is directly related to the applied dose.^4,10,14,23,31,37^

**Table 5:** Antimicrobial Performance of Pure and Doped Silver Phosphate and Reported in the Literature^a^ ^b^

		MIC (μgmL^–1^)/Sample					
Bacteria	Gram group	AgP	AgP:0.5Co	AgP:1Co	AgP:3Co	AgP:5Co	ref.
*Staphylococcus aureus* *—ATCC 25922*	Gram-positive	41.29 ± 0.07	48.96 ± 0.21	20.45 ± 0.13	41.48 ± 0.01	41.46 ± 0.04	TS
*Staphylococcus aureus* *—CI 9393*	Gram-positive	13.42 ± 0.05	13.28 ± 0.13	19.74 ± 0.04	12.93 ± 0.03	12.81 ± 0.01	TS
*Enterococcus faecalis* *- ATCC 19433*	Gram-positive	44.03 ± 0.09	26.14 ± 0.16	20.53 ± 0.06	43.18 ± 0.06	42.78 ± 0.13	TS
*Staphylococcus epidermidis* *- ATCC 12220*	Gram-positive	41.39 ± 0.06	15.91 ± 0.05	20.64 ± 0.05	13.31 ± 0.05	13.33± 0.26	TS
*Escherichia coli* *- ATCC 29214*	Gram-negative	18.9 ± 0.09	10.65 ± 0.02	5.50 ± 0.14	10.13 ± 0.08	9.99 ± 0.04	TS
*Klebsiella pneumoniae* *- ATCC 4352*	Gram-negative	9.93 ± 0.08	9.89 ± 0.02	9.96 ± 0.08	20.4 ± 0.05	20.44 ± 0.05	TS
*Pseudomonas aeruginosa* *- ATCC 27853*	Gram-negative	20.35 ± 0.02	20.26 ± 0.02	10.11 ± 0.01	41.28 ± 0.01	41.68 ± 0.07	TS
*Klebsiella pneumoniae* *- CI 230*	Gram-negative	84.2 ± 0.04	38.38 ± 0.01	48.25 ± 0.07	48.67 ± 0.11	48.72 ± 0.05	TS
*Staphylococcus aureus*	Gram-positive	62.5					^4^
*Escherichia coli*	Gram-negative	62.5					^4^
*Staphylococcus aureus*	Gram-positive	31.25					^44^
*Escherichia coli*	Gram-negative	31.25					^44^
*Pseudomonas aeruginosa*	Gram-negative	0.156					^30^

a Values are Expressed as Mean ± Standard Deviation.

b **Legend:** ref. = reference; CI = Clinical isolated; TS = This Study; ATCC = American Type Culture Collection; SD = standard deviation.

In general, it is observed that doping with cobalt ions significantly influences the minimum inhibitory concentration (MIC) values, although this effect varies depending on the bacterial species. For Gram-positive bacteria, such as *Staphylococcus aureus* ATCC 25922, MIC values remain relatively high (≈ 41–49 μgmL^–1^) for most formulations, except for AgP:1Co, which shows a significant reduction (20.45 μgmL^–1^), improving antimicrobial activity at a specific doping ratio. A similar trend is observed for *Enterococcus faecalis* ATCC 19433, where AgP:0.5Co and AgP:1Co present reduced values compared to the pure material.

For *Staphylococcus epidermidis* ATCC 12220, a marked reduction in MIC is observed in the doped formulations, especially for AgP:0.5Co and AgP:3Co, with values (∼13–16 μgmL^–1^) significantly lower than pure AgP (∼41 μgmL^–1^), highlighting a strong positive effect of doping. In contrast, for the clinical strain of *Staphylococcus aureus* (3933), MIC values are generally lower (≈12–19 μgmL^–1^) and show little variation among formulations, indicating high intrinsic susceptibility of this strain.

Among Gram-negative bacteria, a generally higher sensitivity to the formulations is observed. *Escherichia coli* ATCC 29214 shows a pronounced reduction in MIC with doping, reaching the lowest value for AgP:1Co (5.5 μgmL^–1^), indicating a relevant synergistic effect. Similarly, *Pseudomonas aeruginosa* ATCC 27853 exhibits the lowest MIC for AgP:1Co (10.11 μgmL^–1^), whereas higher dopant concentrations (3 and 5%) result in increased MIC values, suggesting that excessive doping levels may impair efficacy.

For *Klebsiella pneumoniae* ATCC 4352, MIC values are relatively low and show little variation (∼9–20 μgmL^–1^), indicating good antimicrobial activity regardless of the formulation. In contrast, the clinical strain of *K. pneumoniae* (230) presents significantly higher values, especially for pure AgP (84.2 μgmL^–1^), with partial reduction in the doped formulations (∼38–48 μgmL^–1^), highlighting greater resistance and reinforcing the importance of strain variability.

The comparison with literature data,^4,29,50^ reveals that the materials developed in this study show competitive or superior performance in several cases. For example, reported values for *Staphylococcus aureus* and *Escherichia coli* (∼31–62.5 μgmL^–1^) are generally higher than those obtained for the doped formulations in this work. However, an extremely low value for *Pseudomonas aeruginosa* (0.156 μgmL^–1^) reported in the literature indicates that even more efficient systems exist for this particular species.

In summary, the bacterial panel evaluated in this study comprised both Gram-positive (*Staphylococcus aureus*, *Staphylococcus epidermidis*, and *Enterococcus faecalis*) and Gram-negative (*Escherichia coli*, *Klebsiella pneumoniae*, and *Pseudomonas aeruginosa*) species, including reference strains and clinical isolates. These microorganisms exhibit distinct morphological and physiological characteristics that directly influence their susceptibility to antimicrobial agents. Gram-positive bacteria possess a thick peptidoglycan layer and lack an outer membrane, generally facilitating the interaction of antimicrobial compounds with the cell wall and cytoplasmic membrane.^16,44^ In contrast, Gram-negative bacteria present a more complex cell envelope, characterized by a thin peptidoglycan layer surrounded by an outer membrane rich in lipopolysaccharides, which acts as an additional permeability barrier and contributes to their intrinsic resistance.^61–63^ Furthermore, species such as *P. aeruginosa* and *K. pneumoniae* are known for their ability to form biofilms, express multidrug efflux pumps, and produce resistance-associated enzymes, making them particularly difficult to control.^64,65^ Despite these physiological defenses, the silver phosphate-based materials exhibited inhibitory activity against both Gram-positive and Gram-negative bacteria, as evidenced by the MIC values obtained. The enhanced susceptibility observed for some strains, particularly *E. coli* and *S. epidermidis*, may be associated with the ability of silver-containing materials to disrupt membrane integrity, induce oxidative stress through reactive oxygen species generation, and interfere with essential cellular processes. Moreover, the lower MIC values observed for several doped samples compared with pure AgP suggest that metal incorporation can modify the physicochemical properties of silver phosphate, enhancing its antimicrobial.

Figure 12 summarizes the proposed mechanisms involved in the photocatalytic and antimicrobial activities of Co-doped Ag_3_PO_4_.

**Figure 12:**
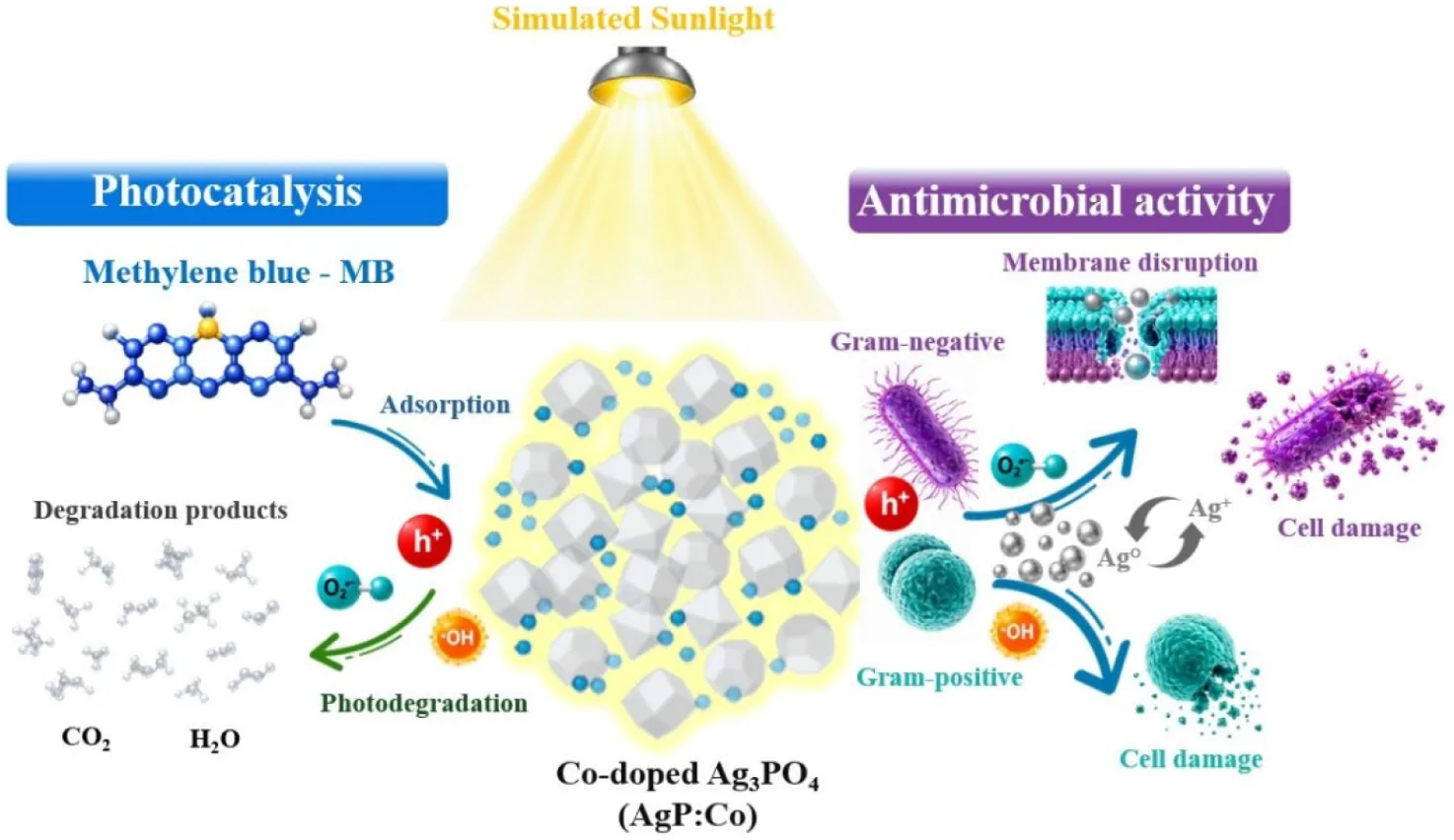
Schematic illustration of the proposed photocatalytic and antimicrobial mechanisms of Co-doped Ag_3_PO_4_ under simulated sunlight, highlighting MB adsorption and oxidation by *h* ^+^,*HO* ^•^, and $O_{2}^{•-}$ , as well as bacterial membrane disruption, oxidative stress, and Ag^+^ release.

Under simulated solar irradiation, photons with energies equal to or greater than the semiconductor bandgap (2.41 eV for AgP:3Co) promote electrons from the valence band to the conduction band, generating photogenerated electrons (*e* ^–^) and holes (*h* ^+^).^18^ Therefore, the Co incorporation modifies the local coordination and electronic structure of Ag_3_PO_4_ through lattice distortion, Ag-site vacancies, and Co-related electronic states, as supported by the Rietveld-refinement, Raman, DRS, PL, and XPS results.^32^ At an optimum Co content, these states may facilitate charge trapping and interfacial transfer, limiting immediate recombination and increasing the availability of charge carriers for surface reactions. This interpretation is consistent with the superior photocatalytic performance of AgP:3Co. Conversely, excessive Co incorporation may generate deep trapping states that favor nonradiative recombination, explaining the lower activity of AgP:5Co despite its stronger PL quenching.

During photocatalysis, MB molecules are initially adsorbed onto the catalyst surface and subsequently oxidized by photogenerated holes and reactive oxygen species. The scavenger experiments indicated that *h* ^+^ and *HO* ^•^ are the principal oxidative species, whereas $O_{2}^{•-}$ provides a secondary and composition-dependent contribution.^10^ Conduction-band electrons can react with dissolved oxygen to form $O_{2}^{•-}$ , while holes can directly oxidize adsorbed MB molecules or participate in the generation of *HO* ^•^ from surface hydroxyl groups or water.^29^ These species attack the dye chromophore, resulting in decolorization and the formation of smaller degradation products. Because complete degradation was not directly demonstrated, the final products are represented as degradation intermediates rather than unequivocally as CO_2_ and H_2_O.^36^

The antimicrobial mechanism is likely governed by the combined effects of released Ag^+^ ions, direct material–cell contact, and possible oxidative stress. Ag^+^ can interact with negatively charged cell-envelope components, membrane proteins, and intracellular biomolecules, disrupting membrane permeability and essential metabolic processes.^30^ Surface contact with Ag_3_PO_4_ particles may further damage the bacterial envelope. In addition, Co-induced changes in surface oxygen species and defect chemistry may favor the formation of reactive species capable of oxidizing lipids, proteins, and other cellular components. These processes can lead to loss of membrane integrity, leakage of intracellular material, and ultimately bacterial inactivation. Differences between Gram-positive and Gram-negative responses may arise from their distinct cell-wall structures and strain-dependent resistance mechanisms.^66^

The presence of surface Ag^0^ identified by XPS and the postreaction structural changes observed by XRD indicate that partial reduction of Ag^+^ can occur during irradiation. Metallic Ag nanoparticles may contribute to charge trapping and antimicrobial activity; however, their formation also represents the initial stage of Ag_3_PO_4_ photocorrosion. The improved activity retention of AgP:3Co over five cycles suggests that cobalt incorporation limits, but does not completely prevent, this transformation. Overall, the proposed mechanism indicates that the performance of Co-doped Ag_3_PO_4_ arises from the interaction among Co-induced defect states, charge-carrier utilization, reactive-species generation, Ag^+^ release, and catalyst–cell contact. Nevertheless, the antimicrobial oxidative-stress pathway remains a literature-supported proposal because ROS generation was not directly measured during the bacterial assays.

## 4 Conclusion

Pristine and Co-doped Ag_3_PO_4_ photocatalysts containing nominal Co concentrations of 0.5–5 mol % were successfully prepared by the hydrothermal method. XRD and Rietveld refinement confirmed preservation of the cubic Ag_3_PO_4_ structure without detectable secondary crystalline phases in the as-prepared samples. The variations in lattice parameters, unit-cell volume, crystallite size, and Raman-active modes were consistent with Co-induced local distortion and defect formation. SEM and TEM revealed irregular, quasi-spherical, and agglomerated particles whose morphology and apparent size varied with Co concentration. EDX and ICP-OES confirmed the presence of cobalt, with ICP-OES providing the most reliable bulk quantification, while XPS demonstrated Co-containing surface environments and changes in the chemical states of Ag and O. The XPS results also indicated Ag-deficient, oxygen-rich surfaces, consistent with possible Ag-site vacancies and surface oxygenated species.

The Co incorporation caused only modest changes in the fundamental optical bandgap, which varied from 2.41 to 2.51 eV. However, additional sub-bandgap absorption features at 1.68 and 1.65 eV were observed for AgP:3Co and AgP:5Co, respectively, suggesting Co-related localized electronic states. The progressive PL quenching confirmed that Co strongly modifies the excited-state relaxation pathways. Nevertheless, the comparison between AgP:3Co and AgP:5Co demonstrated that lower steady-state PL intensity does not necessarily correspond to superior photocatalytic activity, because excessive defect formation may favor nonradiative recombination. BET surface areas remained low, ranging from 0.953 to 5.608 m^2^ g^–1^, and did not correlate directly with photocatalytic performance, indicating that activity was governed primarily by the combined electronic, defect-related, and interfacial properties rather than by surface area alone.

The AgP:3Co exhibited the best photocatalytic performance, achieving 93.26% methylene-blue removal after 20 min under simulated solar irradiation, with 120.26 × 10^–3^ min^–1^ and *t*_1/2_ = 5.76 min. Scavenger experiments identified photogenerated holes and hydroxyl radicals as the principal oxidative species, with a secondary contribution from superoxide radicals. AgP:3Co also retained approximately 87% activity after five cycles, compared with approximately 78% for pristine AgP. Postreaction XRD and TEM indicated that Co incorporation improved structural and morphological stability and reduced, although did not completely suppress, photocorrosion. Finally, all materials exhibited antimicrobial activity, with inhibition and MIC responses depending on both Co concentration and bacterial strain. Overall, 3 mol % Co provided the most favorable balance among defect chemistry, charge-carrier utilization, surface reactivity, photocatalytic efficiency, antimicrobial performance, and operational stability, establishing AgP:3Co as the most promising composition investigated.

## Supplementary Material



## Notes

characterizations of: (i) Characterization techniques adopted in this study; (ii) transmission electron microscopy (TEM) of pure and doped silver phosphate before and after the photocatalytic experiments; (iii) N_2_ adsorption/desorption hysteresis; (iv) Band potentials plot for valence and conduction bands; and (v) particle size distribution (PDF)
