# Supplementary material for: Photocatalytic and Antimicrobial Properties of Cobalt-Doped Ag3PO4 Synthesized by Hydrothermal Method
Source: ACS Omega. 2026 Sep 30;11(40):61310–34. doi: 10.1021/acsomega.6c06289 (PMC13670795; doi:10.1021/acsomega.6c06289)
Supplement: Supplementary file 1 [file ao6c06289_si_001.pdf]

# Photocatalytic and antimicrobial properties of cobalt-doped $Ag_3PO_4$ synthesized by hydrothermal method

Daniel Pais Ospina[1,2], Yurimiler Leyet Ruiz[3], David Comedi[1,2], Ronaldo Santos da Silva[4], Simone Ramos de Castro[5], Jéssica Araújo Marques[7], Rubens Lucas de Freitas Filho[8], Marcos A. Bolson[9], Ézio Sargentini-Júnior[9], Lázaro Miranda Carvalho[10], Oscar Marin-Ramirez[1,2] and Francisco Xavier Nobre[5,6*]

[1]*Nanoproject – Laboratorio de Física del Sólido y Laboratorio de Nanomateriales, Departamento de Física, Facultad de Ciencias Exactas y Tecnología, Universidad Nacional de Tucumán, Avenida Independencia 1800, CP 4000, Tucumán, Argentina.*
[2]*Instituto de Física del Noroeste Argentino (INFINOA), Consejo Nacional de Investigaciones Científicas y Técnicas (CONICET) y Universidad Nacional de Tucumán, Avenida Independencia 1800, CP 4000, Tucumán, Argentina.*
[3]*Laboratório de Processamento de Materiais Tecnológicos (LPMAT), Departamento de Ciências e Engenharia de Materiais, Universidade Federal do Amazonas, AM, Manaus, 69067-005, Brazil*
[4]*Department of Physics, Federal University of Sergipe, São Cristóvão, SE, 49100-000, Brazil.*
[5]*Departamento de Química, Alimentos e Meio Ambiente (DQA), Instituto Federal de Educação, Ciência e Tecnologia do Amazonas, Campus Manaus Centro (CMC), Amazonas, Manaus, 69020-120, Brazil.*
[5]*Grupo de Recursos Energéticos e Nanomateriais (GREEN), Instituto Federal de Educação, Ciência e Tecnologia do Amazonas, Campus Manaus Centro (IFAM-CMC), AM, Manaus, 6975-351, Brazil.*
[6]*Laboratório de Processamento de Materiais Tecnológicos (LPMAT), Departamento de Ciências e Engenharia de Materiais, Universidade Federal do Amazonas, AM, Manaus, 69067-005, Brazil*
[7]*Centro Multiusuário para Análise de Fenômenos Biomédicos da Universidade do Estado do Amazonas – CMABio, AM, Manaus, 69065-001, Brazil*
[8]*Departamento de Química, ICEx, Universidade Federal de Minas Gerais, UFMG, Belo Horizonte, MG, 31270-901, Brazil*
[9]*Instituto Nacional de Pesquisas da Amazônia, INPA, Manaus, AM, 69084-000, Brazil*
[10]*Instituto Federal de Educação, Ciência e Tecnologia do Piauí, IFPI, Campus José de Freitas, José de Freitas, 64110-000, Brazil*

*Corresponding author: Francisco.nobre@ifam.edu.br (F.X. Nobre)

## Characterization of Materials, Photocatalytic Experiments and Antimicrobial Assays

*X-ray diffraction*

The samples were characterized by X-ray diffraction (XRD) using a Shimadzu diffractometer, XRD 7000 model with copper anode (CuKα = 0.15406 nm) adopting a step of 0.02°, and scan velocity of 2° $min^{-1}$ in the 2θ interval from 10 to 100°. The structural Rietveld refinement was calculated through the public domain Fullprof suit software, version November 2015 for Windows. Therefore, the Thompson-Cox-Hastings pseudo-Voigt * Axial divergence asymmetry function was used in the adjustment of intensity and profile of XRD diffraction peaks, while the background was refined using the linear interpolation. In this way, the lattice parameters (*a*, *b*, *c*, α, β and γ), atomic position (*x*, *y* and *z*), occupation (Occ), and unit cell volume (V) were also refined, which is monitored by R-factors: $\chi^2$, $R_p$, $R_{wp}$ and $R_{exp}$.

*Scanning Electron Microscopy – SEM*

The morphology and surface microstructure of the synthesized samples were examined by scanning electron microscopy (SEM) using a Carl Zeiss Supra 55-VP field-emission scanning electron microscope operated at an accelerating voltage of 15 kV. The SEM analysis provided detailed information regarding particle size distribution, agglomeration behavior, and surface texture of the obtained materials.

*Vibrational Raman Spectroscopy*

The vibrational properties of the synthesized materials were investigated by confocal micro-Raman spectroscopy using a Bruker Senterra spectrometer equipped with a Peltier-cooled CCD detector and an Olympus BX50 microscope. Raman spectra were acquired at room temperature under excitation with a 785 nm laser line operating at an output power of 5 mW.

*UV-vis by diffuse reflectance spectroscopy*

The optical properties of the synthesized samples were investigated by UV–Vis diffuse reflectance spectroscopy (DRS) using a Shimadzu UV-2600i spectrophotometer equipped with a diffuse reflectance accessory. The spectra were recorded in the wavelength range of 190 to 800 nm at room temperature, employing the barium sulphate ($BaSO_4$) Sigma-Aldrich, purity ≥ 99.9%) as the reflectance standard. The obtained diffuse reflectance data were used to evaluate the light absorption behavior and estimate the optical band gap energy of the materials.

*Photoluminescence spectroscopy*

Photoluminescence (PL) spectroscopy measurements were performed using an iHR320 Multimodal Horiba spectrofluorometer. The emission spectra were recorded at room temperature in the wavelength range of 400 to 750 nm under an excitation wavelength of 375 nm.

*Transmission electron microscopy (TEM)*

The morphology and particle dimensions of LiNb were examined by transmission electron microscopy (TEM). The particle suspension was deposited onto a glow-discharged, carbon-coated 200-mesh copper grid (Excel support film, Nisshin EM Co.). Excess liquid was removed using filter paper, after which the grid was allowed to dry completely. TEM micrographs were obtained using a JEM-1400Flash electron microscope (JEOL).

*X-ray photoelectron spectroscopy (XPS)*

The surface elemental chemical states were characterized by X-ray photoelectron spectroscopy (XPS) using a Scienta Omicron ESCA+ spectrometer. The instrument was equipped with a monochromator, an Al Kα radiation source (eV), an EAC-2000 high-performance hemispherical electron-energy analyzer, and a low-energy electron source for charge neutralization of insulating samples. Measurements were collected at an electron take-off angle of 54.7° from an analyzed area of 200 $\mu m^2$. The spectra were processed using a standard Shirley background, and their binding-energy scale was calibrated by setting the C 1s signal to 284.6 eV.

*The $N_2$ Adsorption/Desorption*

Nitrogen physisorption measurements were conducted at −196 °C using an Autosorb-1 analyzer (Quantachrome Instruments, USA) over a relative pressure ($P/P_0$) range of 0.01–1.00. Before analysis, approximately 200 mg of each sample was degassed under vacuum at 140 °C for 12 h. The specific surface area was calculated using the Brunauer–Emmett–Teller (BET) method, while the pore-size distribution was determined by the Barrett–Joyner–Halenda (BJH) method. The total pore volume was estimated from the amount of nitrogen adsorbed at P/P0 = 0.95. Experimental data acquisition and processing were performed using ASiQwin software, version 5.21.

*Analytical measurement of silver and cobalt by ICP-OES*

The Ag and Co concentrations in the samples were determined by inductively coupled plasma optical emission spectrometry (ICP-OES) using a Thermo Scientific iCAP 7600 DUO instrument. The system was equipped with a CETAC U5000AT+ ultrasonic nebulizer and an ASX-520 autosampler. High-purity argon (>99.9999%; White Martins, Manaus, AM, Brazil) was employed for plasma generation, nebulization, and as the auxiliary gas. External calibration curves were prepared by successive dilution of 1000 mg $L^{-1}$ Ag and Co standard stock solutions,

covering a concentration range from 0.5 to 10 mg $L^{-1}$. The emission intensities were monitored at 328.06 nm for Ag and 228.62 nm for Co.

*Photocatalytic Activity*

Photocatalytic activity was evaluated by monitoring the degradation of methylene blue (MB) under simulated solar irradiation. For each experiment, 25 mg of the photocatalyst was suspended in 50 mL of an aqueous MB solution at an initial concentration of 10 mg $L^{-1}$. Before irradiation, the suspension was magnetically stirred in the dark for 10 min to establish adsorption–desorption equilibrium between MB molecules and the photocatalyst surface. The reaction system was then irradiated using an LCS-100 Series Small Area Solar Simulator equipped with a standard AM1.5G filter. The reactor was positioned at a focal distance of 229 mm, corresponding to an incident optical power density of 105 mW $cm^{-2}$. The degradation process was monitored by UV-Vis absorption Shimadzu UV-Vis-NIR 1280 spectrometer. Absorption spectra were recorded at 2 min intervals over a total irradiation period of 20 min.

*Antimicrobial assay*

*In vitro* susceptibility assay

The antibacterial activity of the synthesized samples was evaluated against a panel of Gram-positive bacteria, including *Staphylococcus aureus* ATCC 25922, *Bacillus subtilis* ATCC 23857, *Enterococcus faecalis* ATCC 19433, and *Staphylococcus epidermidis* ATCC 12220, as well as Gram-negative bacteria *Pseudomonas aeruginosa* ATCC 27853, *Escherichia coli* ATCC 29214, and *Klebsiella pneumoniae* ATCC 4352. In addition, resistant clinical isolates, namely *Staphylococcus aureus* clinical strain 9393, *Staphylococcus aureus* clinical strain RIB1, and *Klebsiella pneumoniae* clinical strain 230, kindly provided by the Hospital das Clínicas of the University of Campinas (UNICAMP), were also included in the study.

The antimicrobial susceptibility tests were performed by agar well diffusion method following the guidelines established by the Clinical and Laboratory Standards Institute (CLSI). Initially, each bacterial strain was suspended in 5 mL of Mueller–Hinton broth until reaching a turbidity equivalent to 0.5 McFarland standard. After homogenization, a sterile swab was immersed in the bacterial suspension and pressed against the inner wall of the tube to remove excess inoculum. The microorganisms were then uniformly inoculated onto the surface of Mueller–Hinton agar plates by streaking in three different directions, rotating the plate approximately 60° between each streak to ensure homogeneous distribution.

After inoculation, the plates were allowed to stand for approximately 5 min at room temperature to permit absorption of residual surface moisture. Subsequently, wells were aseptically made in the agar medium, and 25 μL of each sample solution (100 mg $mL^{-1}$) was

introduced into the respective wells. Ciprofloxacin solution (1 $mgmL^{-1}$), prepared in sterile saline, was used as the positive control. The inoculated plates were incubated at 37 °C for 24 h. After the incubation period, the inhibition zones formed around each well were measured using a digital caliper under reflected light, with the plates positioned upside down to improve visualization. All experiments were carried out in triplicate.

*Minimum Inhibitory Concentration MIC*

The minimum inhibitory concentration (MIC) of the synthesized samples was determined by the broth microdilution method according to the Clinical and Laboratory Standards Institute (CLSI) guidelines. The antibacterial assay was carried out against Gram-positive bacteria, namely *Staphylococcus aureus* ATCC 25922, *Bacillus subtilis* ATCC 23857, *Enterococcus faecalis* ATCC 19433, and *Staphylococcus epidermidis* ATCC 12220, as well as Gram-negative bacteria *Pseudomonas aeruginosa* ATCC 27853, *Escherichia coli* ATCC 29214, and *Klebsiella pneumoniae* ATCC 4352. Resistant clinical isolates, including *Staphylococcus aureus* clinical strain 9393, *Staphylococcus aureus* clinical strain RIB1, and *Klebsiella pneumoniae* clinical strain 230, were also evaluated.

The bacterial inoculum was prepared by suspending freshly cultured colonies in sterile saline solution, and the turbidity was adjusted to the 0.5 McFarland standard, corresponding to approximately CFU $mL^{-1}$. The material suspensions were subjected to twofold serial dilution in Mueller–Hinton broth using sterile 96-well microplates. The assay included a bacterial growth control containing culture medium and inoculum, a sterility control containing culture medium only, a positive antimicrobial control containing ciprofloxacin, and a killed-cell control consisting of a bacterial suspension previously treated with ethanol. The latter was used exclusively as a no-growth reference and to distinguish bacterial proliferation from any background turbidity caused by the suspended materials. Ethanol was not added to the wells containing the evaluated materials and therefore did not interfere with the determination of the minimum inhibitory concentration (MIC).

After addition of the inoculum, the microplates were incubated at 37 °C for 24 h. Subsequently, 40 μL of 0.01% resazurin solution was added to each well as a viability indicator, followed by an additional incubation period of 3 h. Bacterial growth was initially evaluated by visual inspection based on the colorimetric change of the resazurin dye. Thereafter, the optical density ($OD_{600}$) of each well was measured using a microplate reader at 600 nm.

The mean $OD_{600}$ values were plotted as a function of sample concentration after subtraction of the average absorbance obtained for the sterility control. The MIC value was defined as the lowest sample concentration capable of inhibiting visible bacterial growth, showing no statistically significant difference in comparison with sterility control. Statistical comparisons

among $OD_{600}$ values were performed by two-way analysis of variance (ANOVA) considering a 95% confidence level.

**Chapter Figure**

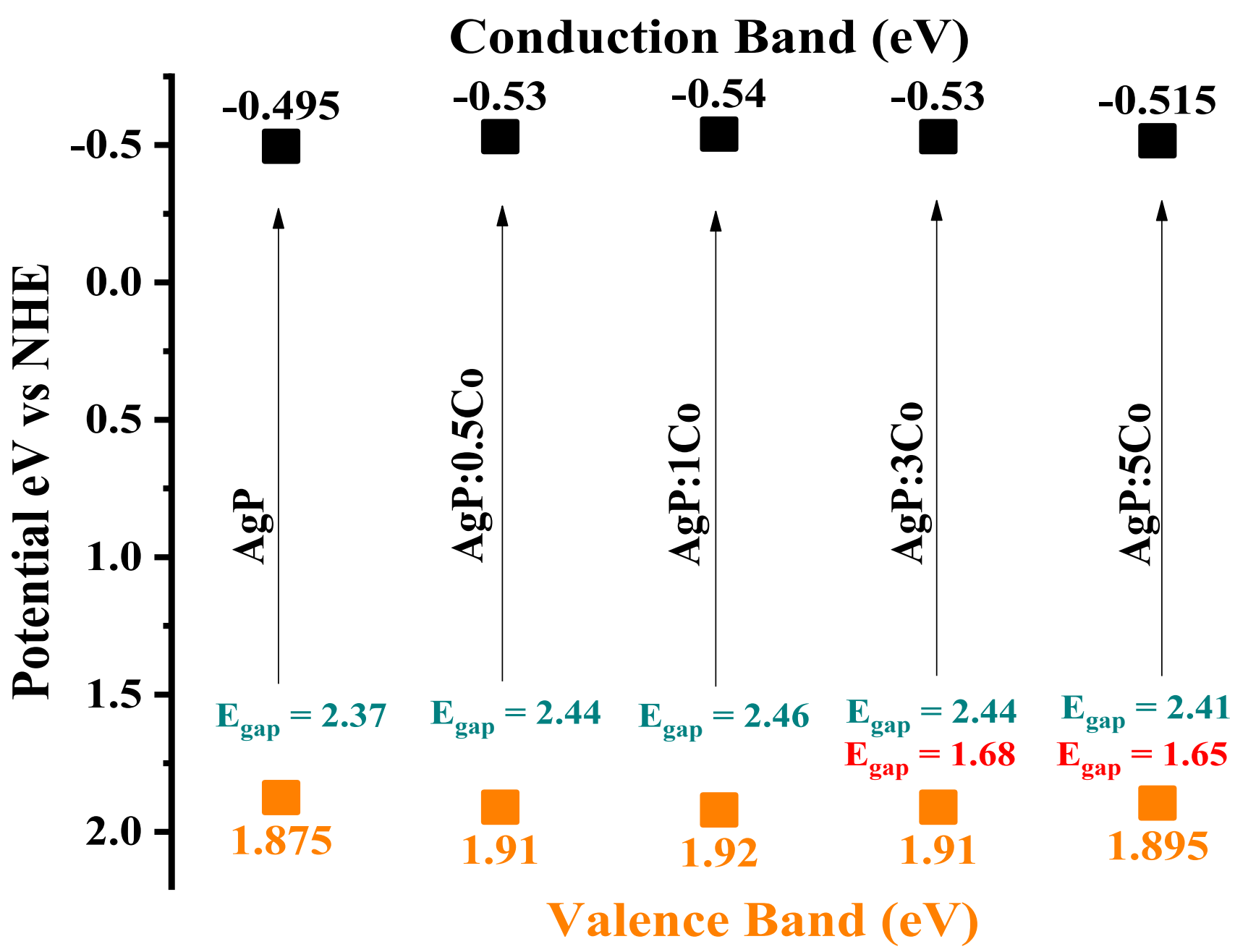


**Figure S1.** Potentials for valence and conduction bands of AgP, AgP:0.5Co, AgP:1Co, AgP:3Co, and AgP:5Co samples.

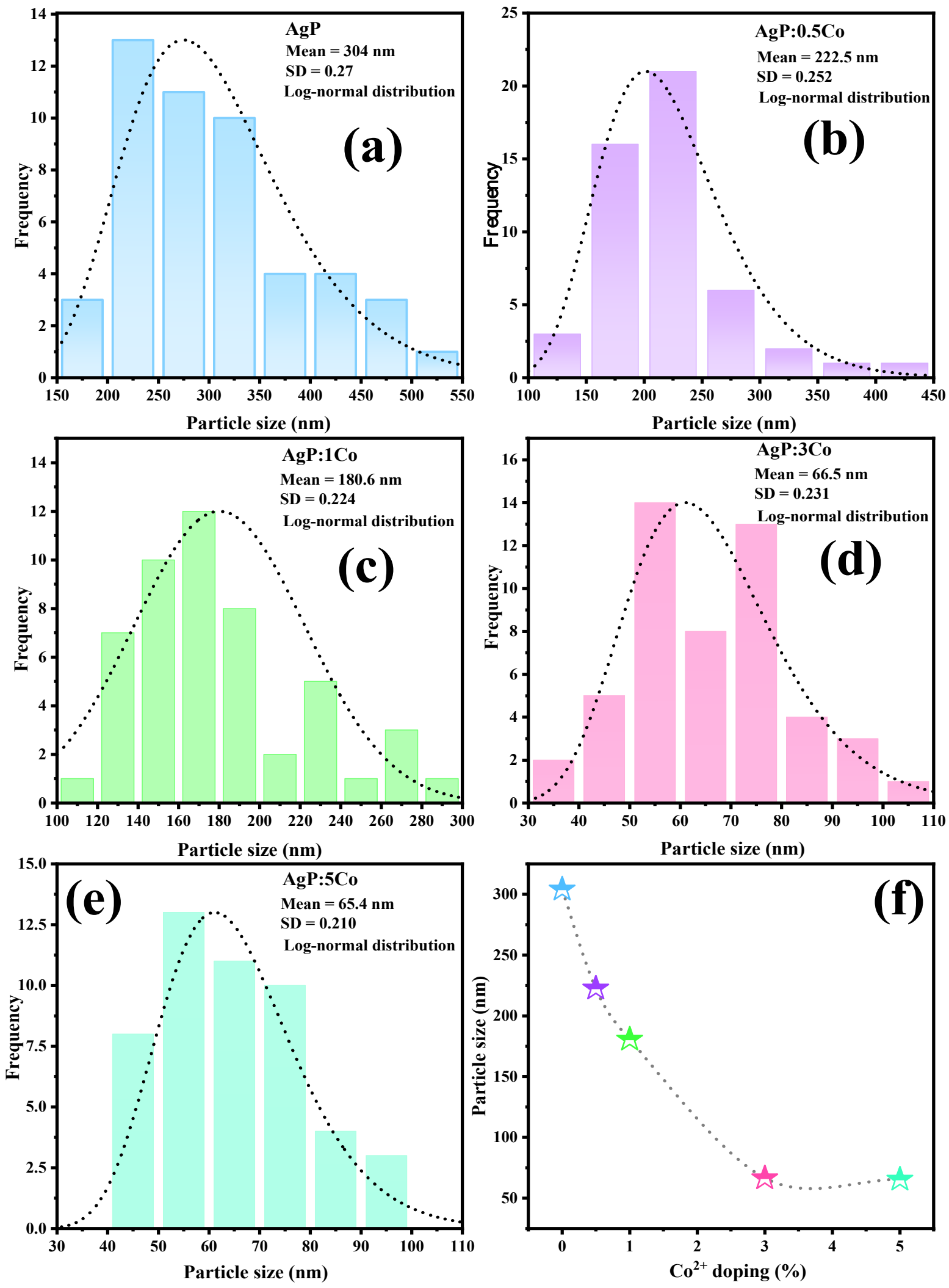


**Figure S2.** particle size distribution for (a) AgP, (b) AgP:0.5Co, (c) AgP:1Co, (d) AgP:3Co, (e) AgP:5Co, and (f) particle size against $Co^{2+}$ doping (%) correlation.

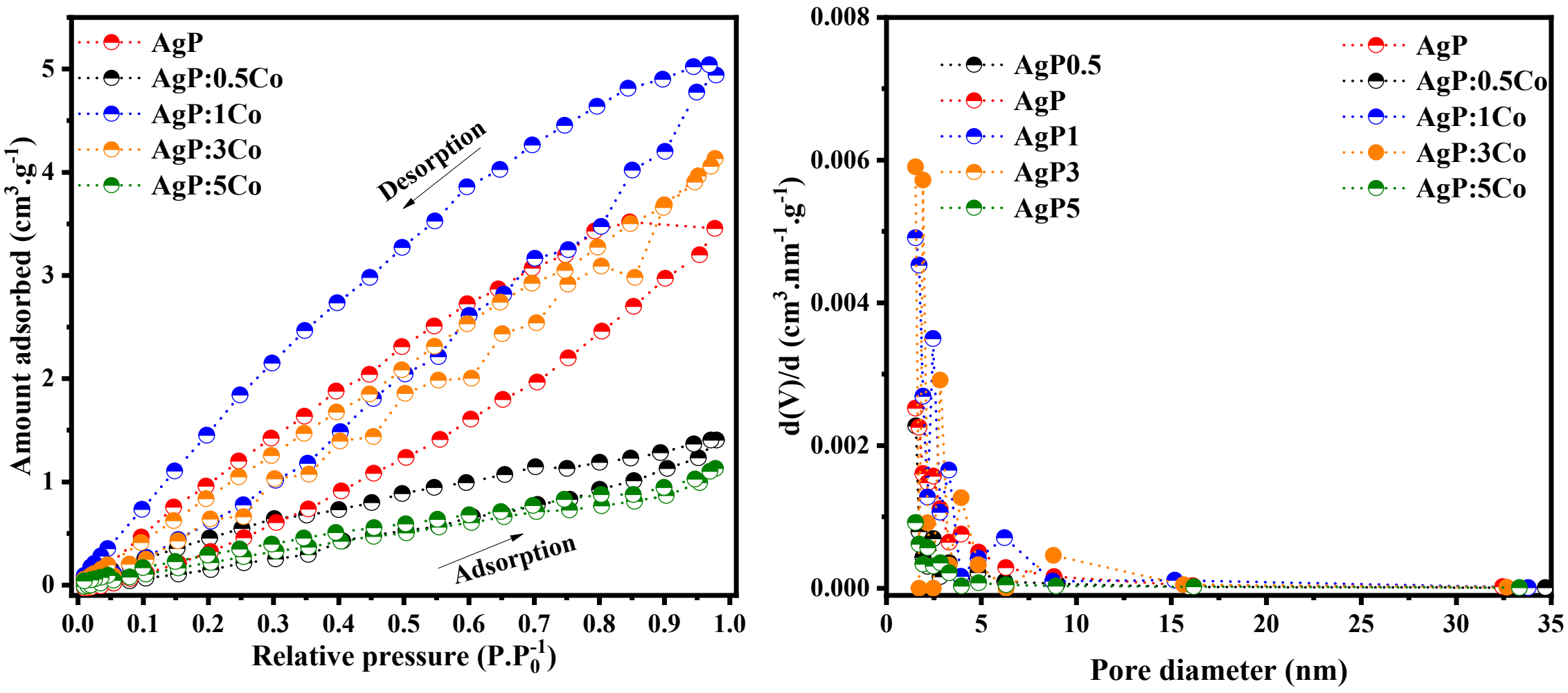


**Figure S3.** Potentials for valence and conduction bands of AgP, AgP:0.5Co, AgP:1Co, AgP:3Co, and AgP:5Co samples.

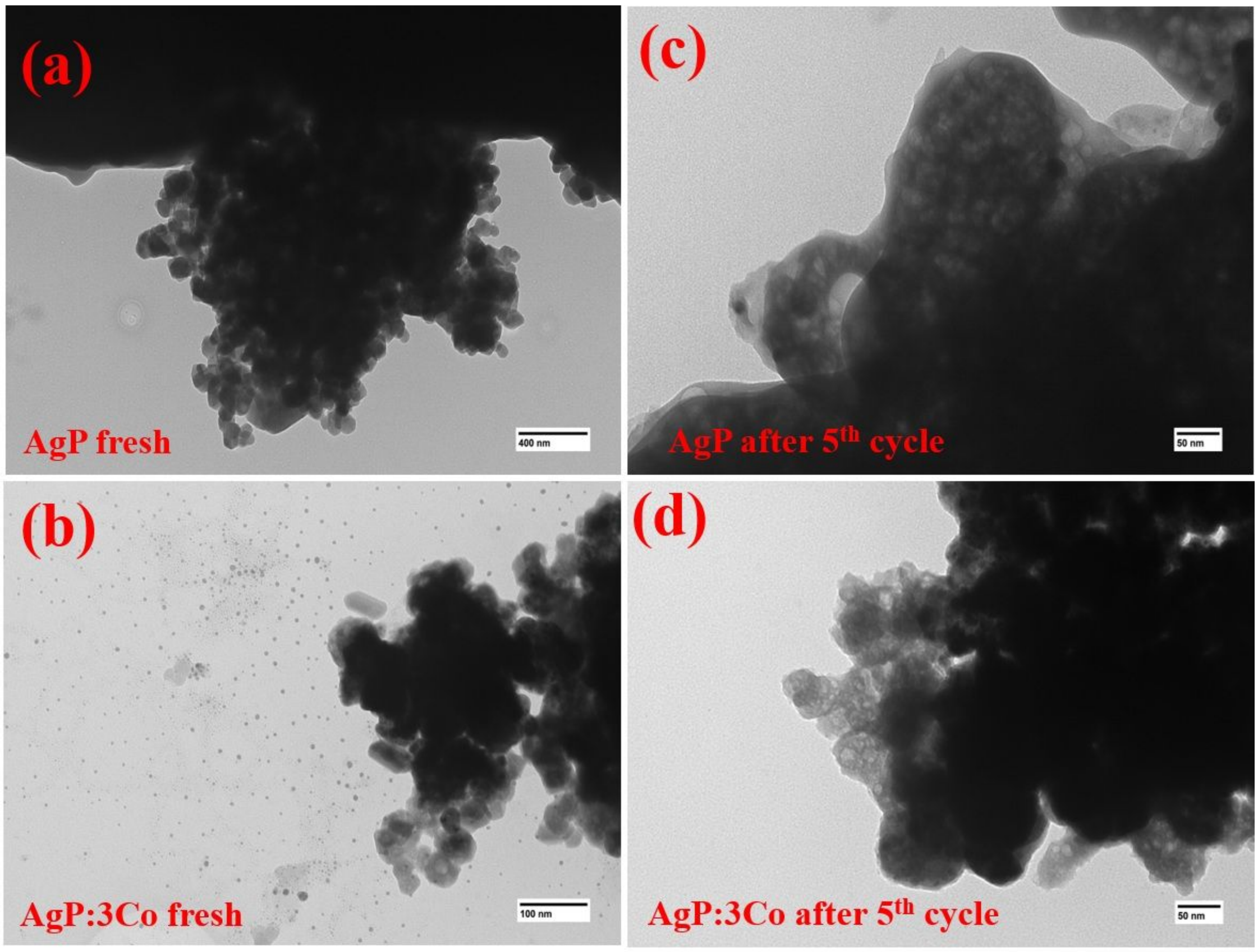


**Figure S4.** TEM images of (a) fresh AgP, (b) fresh AgP:3Co, (c) AgP after the fifth photocatalytic cycle, and (d) AgP:3Co after the fifth photocatalytic cycle**.**

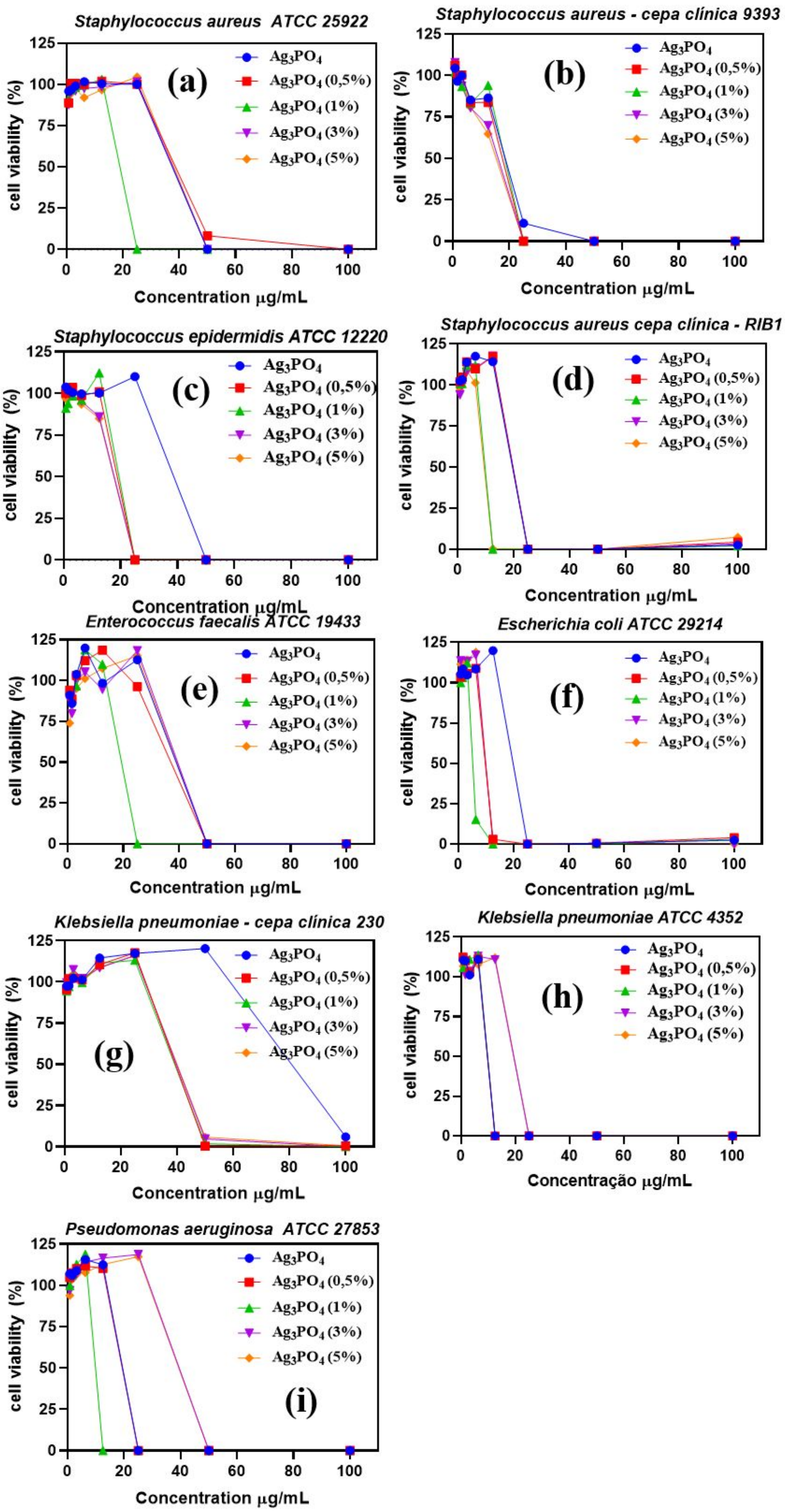


**Figure S5.** Cell viability of reference and clinical bacterial strains after exposure to pristine $Ag_3PO_4$ (AgP) and Co-containing samples (AgP:0.5Co, AgP:1Co, AgP:3Co, and AgP:5Co) at different concentrations: (a) *Staphylococcus aureus* ATCC 25922; (b) *Staphylococcus aureus*

clinical isolate 9393; (c) *Staphylococcus epidermidis* ATCC 12220; (d) *Staphylococcus aureus* clinical isolate RIB1; (e) *Enterococcus faecalis* ATCC 19433; (f) *Escherichia coli* ATCC 29214; (g) *Klebsiella pneumoniae* clinical isolate 230; (h) *Klebsiella pneumoniae* ATCC 4352; and (i) *Pseudomonas aeruginosa* ATCC 27853. Cell viability is expressed as a percentage relative to the untreated control.
